# Fate and preservation of the Late Pleistocene cave bears from Niedźwiedzia Cave in Poland, through taphonomy, pathology, and geochemistry

**DOI:** 10.1038/s41598-024-60222-3

**Published:** 2024-04-29

**Authors:** Adrian Marciszak, Paweł Mackiewicz, Ryszard K. Borówka, Chiara Capalbo, Piotr Chibowski, Michał Gąsiorowski, Helena Hercman, Bernard Cedro, Aleksandra Kropczyk, Wiktoria Gornig, Piotr Moska, Dariusz Nowakowski, Urszula Ratajczak-Skrzatek, Artur Sobczyk, Maciej T. Sykut, Katarzyna Zarzecka-Szubińska, Oleksandr Kovalchuk, Zoltán Barkaszi, Krzysztof Stefaniak, Paul P. A. Mazza

**Affiliations:** 1 Department of Palaeozoology, University of Wrocław, Wrocław, Poland; 2 Department of Bioinformatics and Genomics, University of Wrocław, Wrocław, Poland; 3 Institute of Marine and Environmental Sciences, Szczecin University, Szczecin, Poland; 4 Department of Earth Sciences, University of Florence, Florence, Italy; 5 Faculty of Biology, Biological and Chemical Research Centre, University of Warsaw, Warsaw, Poland; 6 Institute of Geological Sciences, Polish Academy of Sciences, Warsaw, Poland; 7 Institute of Marine and Environmental Sciences, University of Szczecin, Szczecin, Poland; 8 Department of Evolutionary Biology and Conservation of Vertebrates, University of Wrocław, Wrocław, Poland; 9 Institute of Physics – Centre for Science and Education, Silesian University of Technology, Gliwice, Poland; 10 Division of Anthropology, Wrocław University of Environmental and Life Sciences, Wrocław, Poland; 11 Institute of Geological Sciences, University of Wrocław, Wrocław, Poland; 12 Center for Ecological Dynamics in a Novel Biosphere (ECONOVO), Department of Biology, Aarhus University, 8000 Aarhus C, Denmark; 13 Department of Archaeology and Heritage Studies, Aarhus University, Moesgård Allé 20, 8270 Højbjerg, Denmark; 14 Mammal Research Institute, Polish Academy of Sciences, Stoczek 1C, 17-230 Białowieża, Poland; 15 National Academy of Sciences of Ukraine, National Museum of Natural History, Kyiv, Ukraine; 16 Department of Agricultural Sciences, John Von Neumann University, Kecskemét, Hungary

**Keywords:** Biogeochemistry, Climate sciences, Ecology, Natural hazards, Diseases, Trauma, Environmental sciences, Environmental impact, Ecology, Biogeochemistry, Ecosystem ecology, Palaeoecology, Biochemistry, Biogeochemistry, Metals

## Abstract

This comprehensive study examines fossil remains from Niedźwiedzia Cave in the Eastern Sudetes, offering detailed insights into the palaeobiology and adversities encountered by the Pleistocene cave bear *Ursus spelaeus ingressus*. Emphasising habitual cave use for hibernation and a primarily herbivorous diet, the findings attribute mortality to resource scarcity during hibernation and habitat fragmentation amid climate shifts. Taphonomic analysis indicates that the cave was extensively used by successive generations of bears, virtually unexposed to the impact of predators. The study also reveals that alkaline conditions developed in the cave during the post-depositional taphonomic processes. Mortality patterns, notably among juveniles, imply dwindling resources, indicative of environmental instability. Skeletal examination reveals a high incidence of forelimb fractures, indicating risks during activities like digging or confrontations. Palaeopathological evidence unveils vulnerabilities to tuberculosis, abscesses, rickets, and injuries, elucidating mobility challenges. The cave’s silts exhibit a high zinc concentration, potentially derived from successive bear generations consuming zinc-rich plants. This study illuminates the lives of late cave bears, elucidating unique environmental hurdles faced near their species’ end.

## Introduction

**Figure 1:**
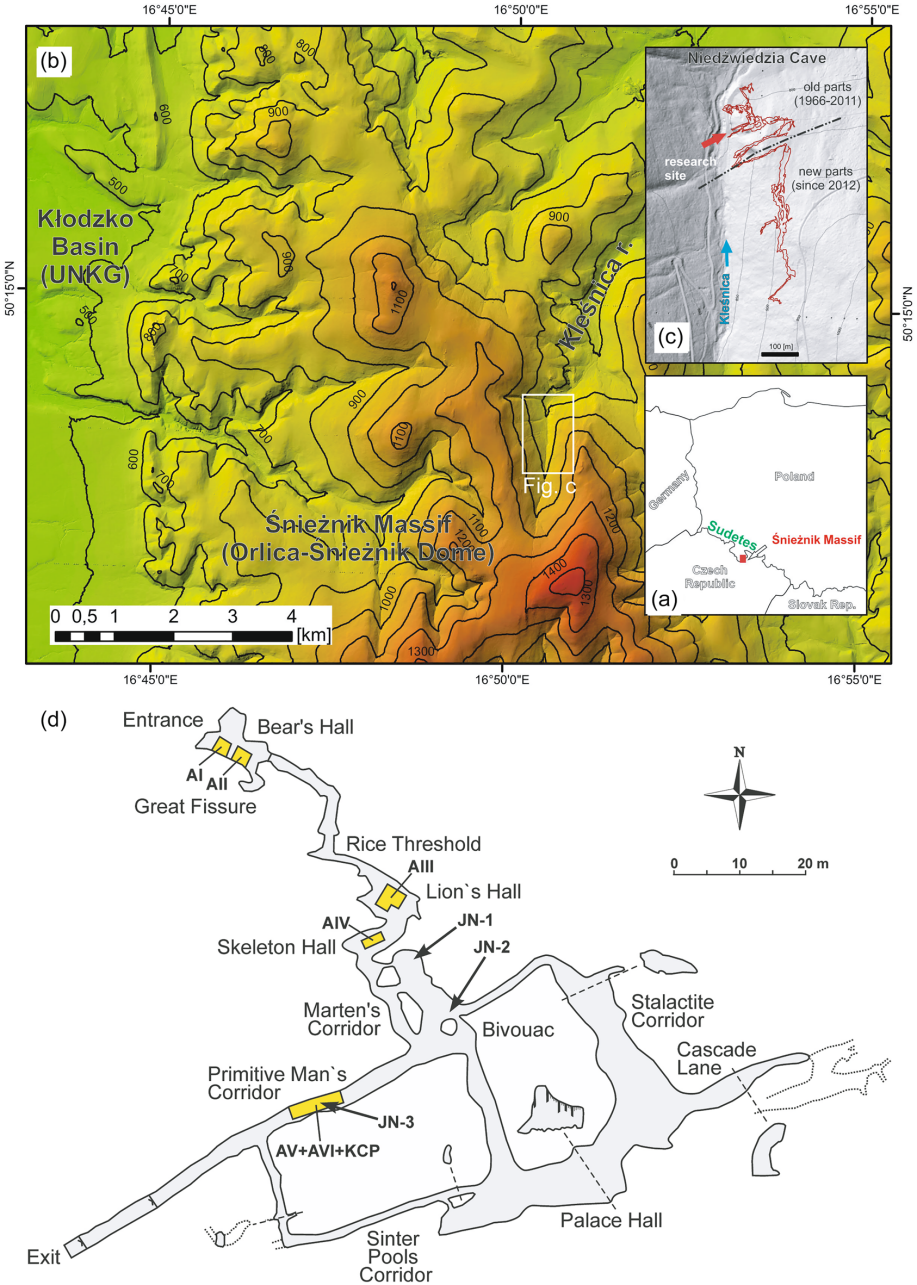
Geographical location of Niedźwiedzia Cave in the Kleśnica River valley, in the Śnieżnik Massif in Poland. The figure displays: (**a**) the cave's position in Poland, (**b**) details of the Śnieżnik Massif region with white outline indicating the cave, (**c**) representation of the 1966–2011 (upper) and post-2012 (lower) cave explorations, and (**d**) cave plan showing the *Ursus spelaeus ingressus* fossil-bearing areas (AI-AVI, KCP) and three silt profiles (JN-1, JN-2, JN-3) sampled for geochemical analyses. Maps (**a**) and (**b**) are based on LiDAR DTM data (source: https://www.geoportal.gov.pl/) and generated in ESRI ArcMap 10.8.2 licensed to the Faculty of Earth Sciences and Environmental Management, University of Wrocław, Poland. Cave passage outlines generated from measurements provided by Kostka^96^.

The Niedźwiedzia (Bear) Cave, nestled in Kleśnica river valley in the Śnieżnik Massif of the Eastern Sudetes in Poland (Fig. 1), has been a focal point of extensive palaeontological scrutiny. Its discovery in 1966, during excavations in a marble quarry, began ongoing explorations that have progressively increased the discovered length of the cave corridors to surpass 5000 m by speleological expeditions conducted between 2011 and 2014. Nevertheless, the total length of the cave corridors is much longer, as indicated by new results from geophysical imaging^1^.

The cave was formed within the marbles dated from Middle Cambrian to Early Ordovician (520–470 Ma)^2^. Their karstification began after the Paleogene basin inversion climax (65–45 Ma)^3^ before its final post-Mid-Miocene uplift^4^. The karstification process in Kleśnica Valley aligned with WSW–ENE trending sub-vertical faults and fractures, with N–S to NNW–SSE steeply dipping foliation playing a subordinate role^5^. The cave’s sections exhibit distinct structural patterns; the southern regions feature elongated N–S passages, while the northern areas demonstrate a grid-like structure (Fig. 1). The earliest passages, presumed to predate the Mid-Pleistocene, witnessed multiple phases of heightened tectonic activity and potential palaeoearthquakes^6^.

The corridors of Niedźwiedzia Cave house a diverse array of allochthonous and autochthonous deposits, including rock debris, fluvial gravels, sands, and dripstones^7^. After its discovery, intensive paleontological investigations were carried out in the middle level II of the cave^5^, leading to the excavation of two faunal assemblages of different ages. The older one dates back to Marine Isotope Stages (MIS) 3–2 and includes: *Sorex araneus*, *Crocidura suaveolens*, *Castor fiber*, *Arvicola terrestris*, *Microtus arvalis*, *Bison priscus, Rupicapra rupicapra, Rangifer tarandus*, *Sus scrofa*, *Gulo gulo*, *Meles meles*, *Martes martes*, *Mustela erminea*, *M. nivalis*, *Canis lupus spelaeus*, *Vulpes lagopus*, *V. vulpes*, *Ursus arctos priscus*, *U. spelaeus ingressus*, and *Panthera spelaea spelaea.* The remains of *U. spelaeus ingressus* comprised around 98.5% of the findings^8^. The remaining 1.5% constitutes other species, demonstrating the diversity in the cave’s historical fauna^8–11^. The remains of the younger fauna (*Eptesicus nilssoni*, *Myotis brandti*, *M. daubentoni*, *M. myotis*, *M. mystacinus*, *M. nattereri*, *M. bechsteini*, *Plecotus auritus*, *Microtus arvalis*, *V. vulpes*, *U. arctos arctos*, *Meles meles*, *Martes martes*, *M. putorius*, *Lynx lynx*, *Felis sylvestris*, *C. elaphus*, *Capreolus capreolus*, *Sus scrofa*) date to MIS 1^8,9,11^.

The importance of this cave lays in the abundance of well-preserved *U. s. ingressus* remains^8^. This subspecies, identified as the largest and most evolved cave bear, presents many distinctive morphological traits^12^. The fossil record, comprising skeletal elements and thousands of isolated teeth, provides valuable insights into their ecological adaptations, suggesting a specialised vegetarian diet^13,14^.

The abundance of well-preserved remains positions this cave as a pivotal reference for morphodynamic analyses in Central European populations. Morphologically akin to the primary population of *U. s. ingressus* from Gamssulzenhöhle (Austria, MIS 3), the specimens recovered from the cave surpass it in size, potentially influenced by factors like Bergman’s rule associated with the more northern location of the site. Morphodynamic investigations reveal a minimal degree of morphological diversity and suggest the population’s relative age during MIS 3 or 4. Radiocarbon dating places the age of *U. s. ingressus* remains between approximately 80–40 ka^15^, signifying their earlier presence in the Sudetes compared to Western Europe. Ancient DNA analysis confirmed these remains as *U. s. ingressus*^15,16^, displaying distinctive haplotypes considerably divergent from those in other European sites, implying the probable isolation of this bear population from others.

The stratigraphic ages derived from current studies indicate that representatives of this species utilised the Niedźwiedzia Cave from MIS 5 through the first half of MIS 3, coinciding with a period marked by significant climate instability, represented by alternating warm and moist with cool-cold periods called Dansgaard–Oeschger (D–O) and Heinrich events (H)^17,18^. These fluctuations resulted in temperature spikes of 5–8 °C during warm D–O intervals^19^. Although occurring irregularly, D–O events recurred every few thousand years, while H events had an approximate 10 000-year separation, lasting about 1–2 ka during MIS 3. *U. s. ingressus* occupied Niedźwiedzia Cave during severe H 5–4 events around 46 and 40 ka and intense D–O 14 and 12 events about 52 and 45 ka, respectively.

*U. s*. *ingressus* vanished during H 4 following H 5, with all cave bears disappearing between 26,100–24,300 cal. years BP at the end of Greenland Stadial 3, culminating in the H 2 peak at 22 ka^20,21^. This subspecies potentially outlasted its relative, *Ursus spelaeus spelaeus*, by about 1000 years, possibly due to a higher adaptability to arid environments and increased morphological variability^22^. Reduced vegetation caused by worsening climate might have prolonged hibernation, heightening vulnerability to human activity and predators. The extensive climatic variations of MIS 5 through MIS 2 offer an opportunity to study Holocene climate evolution and faunal extinctions.

Employing a comprehensive multidisciplinary and interdisciplinary approach, which includes extensive palaeobiological, taphonomic, palaeopathological, and geochemical analyses performed on sediments, along with collagen-based isotopic analyses, and supported by a chronological framework established through radiocarbon and U-Th dating, this study aims to unravel the behavior, interactions, and ecological contexts of *Ursus spelaeus ingressus* populations in Poland. The overarching goal is to provide insights into the fate of one of the last cave bear populations in Central-Eastern Europe and to understand the dynamic evolution of its interconnected ecosystem over time.

## Results

### Palaeobiological and taphonomic analysis

**Figure 2:**
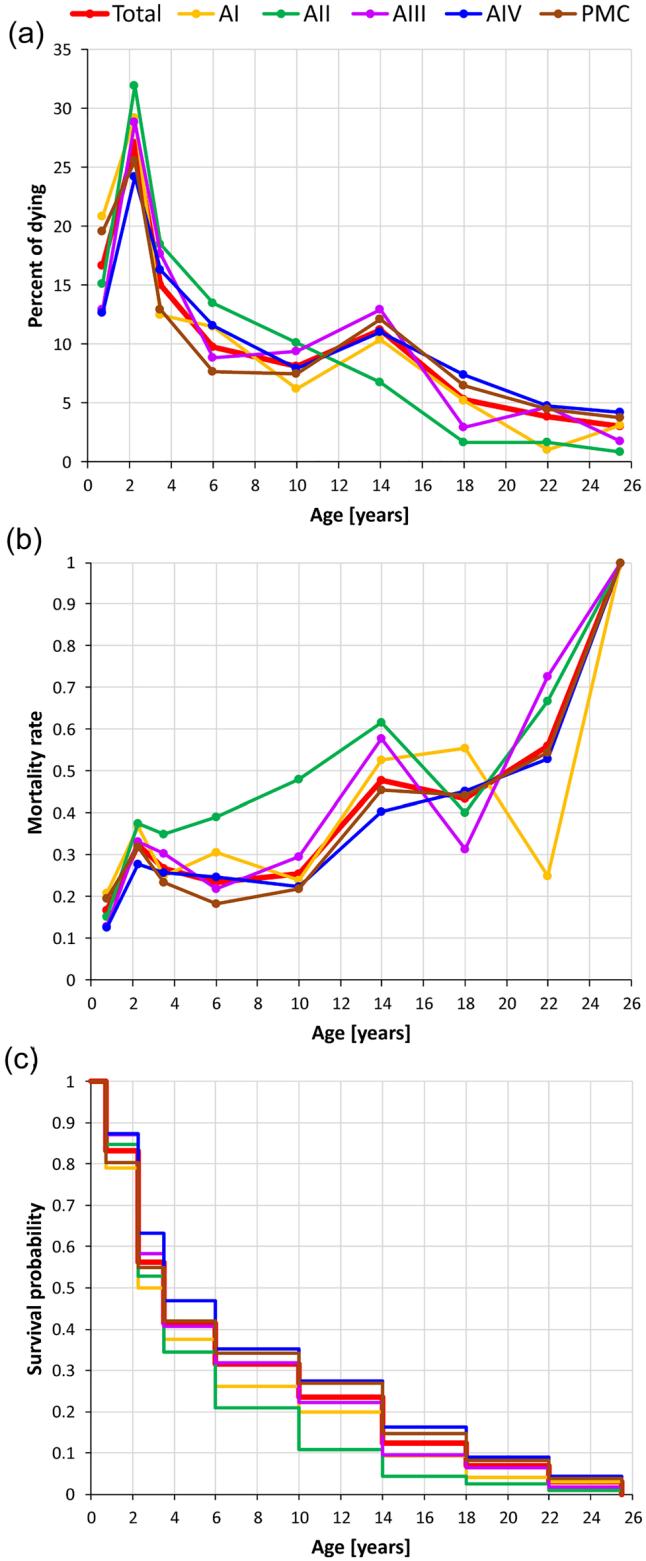
Survival diagrams showing the cave bear demographic patterns based on distinct excavation area and total dataset counts (Total). 'PMC' indicates areas V, VI, and KCP in the Primitive Man’s Corridor. (**a**) Percentage of dead individuals. (**b**) Mortality rates. (**c**) Kaplan–Meier survival curves portraying survival times.

Fisher’s exact test did not refute the hypothesis of equal left and right cheek teeth proportions across 151 layers and excavation areas. When grouped into nine wear stages, 27 of 33 levels showed a positive Spearman’s correlation coefficient in tooth distributions (average coefficient: 0.37). However, only four instances were statistically significant. Despite this, other tests did not reject the hypothesis, suggesting similar left and right tooth distributions in age classes across examined levels. Among 151 levels, MNI distributions based on ontogenetic ages correlated positively with the reference MNI from excavation sites of Niedźwiedzia Cave. The average Spearman’s coefficient was 0.48, with 78 cases above 0.5, and 25 significant. Other tests showed no statistical distinction. The mortality curve depicted a decline: high (13–21%) at stage I (mean age 0.75), peaking at stage II (ca. 2.25 years; Fig. 2a), and ranging 24–32% across areas (27% overall). Minor peaks occurred at stage VI (ca. 14 years), reaching 11%. Notably, area II lacked this peak.

The mortality rate rises irregularly with ontogenetic age (Fig. 2b). After peaking in stage II, most areas and the full dataset plateau in stages III–V, then escalate in stage VI. This trend continues until the final stage, with a slight decline in stage VII (around 18 years). Notably, the trend of area II differs, showing higher rates for stages II–VI. Also, the rate of area I notably drops below others in stage VIII (ca. 22 years).

The Kaplan–Meier survival curves show a similar general trend (Fig. 2c), but area II displays lower survival rates. The steep curves initially indicate higher early-age mortality. Median survival was 2.875 years for area I and 3.5 years for combined data from all sites. No tests detected significant differences between survival curves from individual areas and the global curve.

**Figure 3:**
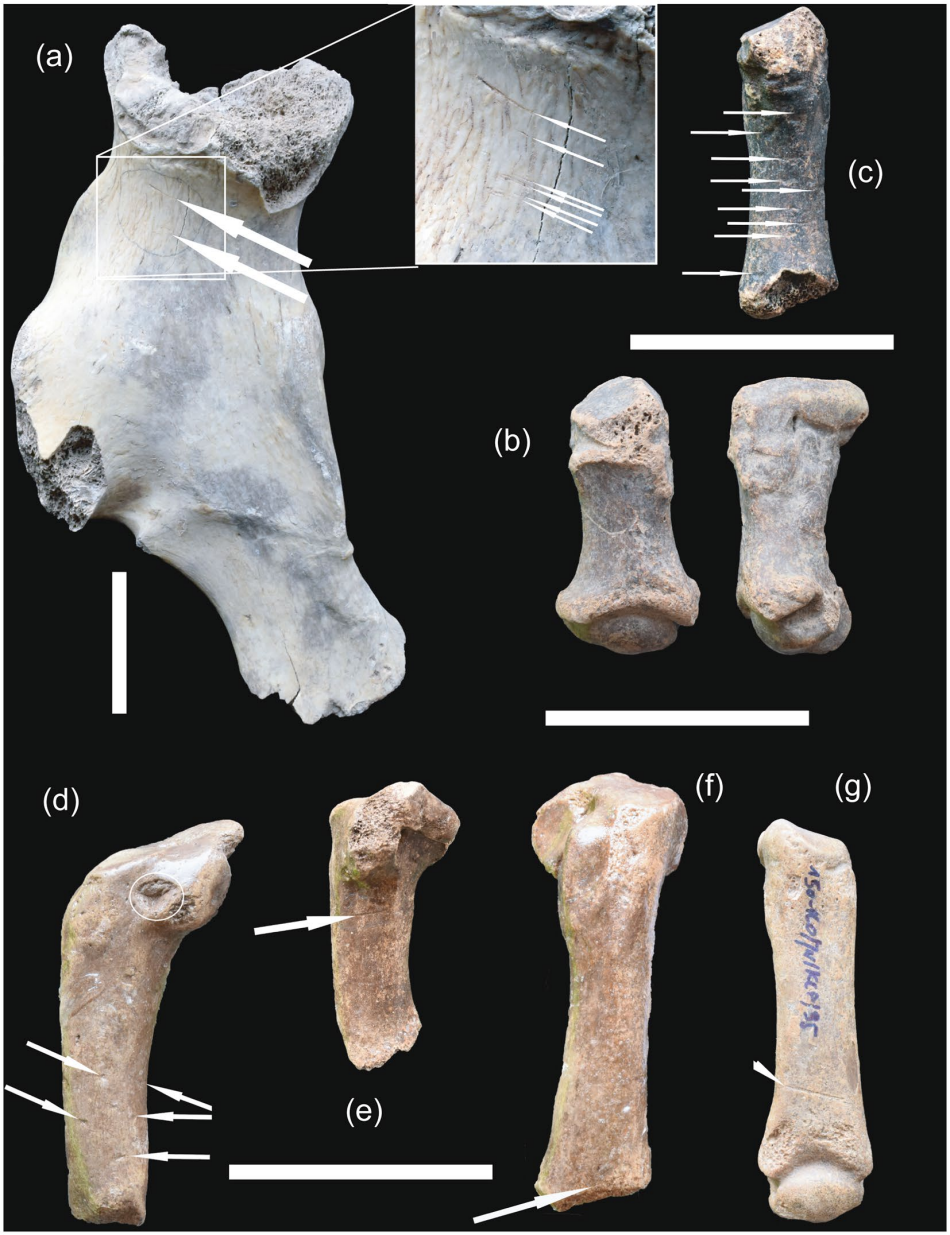
Taphonomic modifications. (**a**) Right coxal bone 12,052 from AV, layer 22, showing parallel, shallow, multiplied scratches created by trampling and a deeper trampling incision (detail). Arrows indicate trampling breakage on pubis and alkaline corrosion evidence on ileum. (**b**) II left brachymetacarpal bone 18,745 from area KCP, with alkaline corrosion evidence on proximal epiphysis and alkaline corrosion on proximal epiphysis. (**c**) II right metatarsal bone 18,954 from KIII, layer 22, featuring a rare instance of isolated tooth puncture marks, along with trampling scratches, grooves, and alkaline corrosion on the proximal epiphysis. (**d**) V left metatarsal bone 2502 from area KCP, with arrows indicating small, isolated, roundish tooth pit marks, and with circle enclosing a larger, triangular tooth pit mark. Trampling pitting and grooves are also present. (**e**) 2624 V left metatarsal bone from area KCP, the arrow indicates a groove of unknown origin with a U-shaped cross-section, jagged edges and proximal epiphysis with alkaline corrosion evidence. (**f**) V left metatarsal bone 2556 from area KCP, with transverse trampling cut and proximal epiphysis with alkaline corrosion evidence. (**g**) II right metatarsal bone 1782 from area KCP arrow indicating a groove with a trapezoidal cross-section, possibly caused by trampling and alkaline corrosion on both epiphyses. Scale bars 5 cm.

The predominant demographic cohort comprises 58.7% juveniles (stages I–III), with 34.4% as prime adults (stages IV–VII) and 7% as old adults (stages VIII–IX) (Fig. 2). High mortality rates in these groups prompted scrutiny via taphonomic, dental micro- and mesowear, and isotopic analyses. Despite bears typically living nearly three decades, the causes of these deaths remain unclear. Tooth wear suggests a plant-based diet, aligning with the results of the stable isotope analysis reported below and with their herbivorous tendencies as seen in similar species^13,14,23–26^. Skeletal remains exhibit extensive chemical corrosion (Fig. 3a-c, e–g), except for teeth. Additionally, a significant number of specimens display distinct manganese oxide patination.

The careful examination of the bone surfaces the Niedźwiedzia Cave specimens detected predominantly shallow marks. These markings rarely occur in discrete groups of parallel incisions; they are more often encountered as scratches or grooves dispersed extensively across the entire cortical surfaces of the specimens, or as isolated signs. Importantly, these marks occur independent of the usable food components of the bones. The right coxal bone 12,052 in Fig. 3 surely held nutritional significance; however, it exhibits only one relatively deeper incision, accompanied by a few superficial scratches. Metapodials, lacking nutritional relevance, display parallel scratches. While it is possible that these are intentional incisions left on these metapodials during skinning procedures, the marks are far too shallow and randomly scattered, making it challenging to conclusively confirm such human activities. In conclusion, a thorough examination does not provide compelling evidence to support categorizing these superficial grooves and scratches as definite human produced cut marks.

### Palaeopathology

Niedźwiedzia fossils show physiological or pathological abnormalities, with five age-related changes in long bones. The analysis revealed 22 pathologically modified instances, potentially from injuries, with 13 linked to inflammation or rickets. Forelimb bones exhibit more frequent abnormalities, indicating susceptibility to injuries in challenging environments. Radiographic exams revealed osteoporosis, osteomalacia, and inflammatory-related bone changes, including degradation and increased density. Histological study indicated inflammation, yet diagnosing conditions like tumours or tuberculosis in palaeopathology demands broader analyses. Three potential tuberculosis cases in an ulna and two vertebrae, abscesses in five long bones, and bone loss in a radius were identified. Rheumatoid conditions, like arthritis, were observed but were infrequent in this sample^27^.

Noted were bone infarctions causing necrosis, periosteal inflammation, and myositis ossificans potentially progressing to a tumour. A likely sarcoma at Niedźwiedzia Cave may have led to death. Two fibrous dysplasia cases and a chondroma (likely spongy osteoma) on a tibia were observed. Ten advanced rickets cases were detected via radiography.

**Figure 4:**
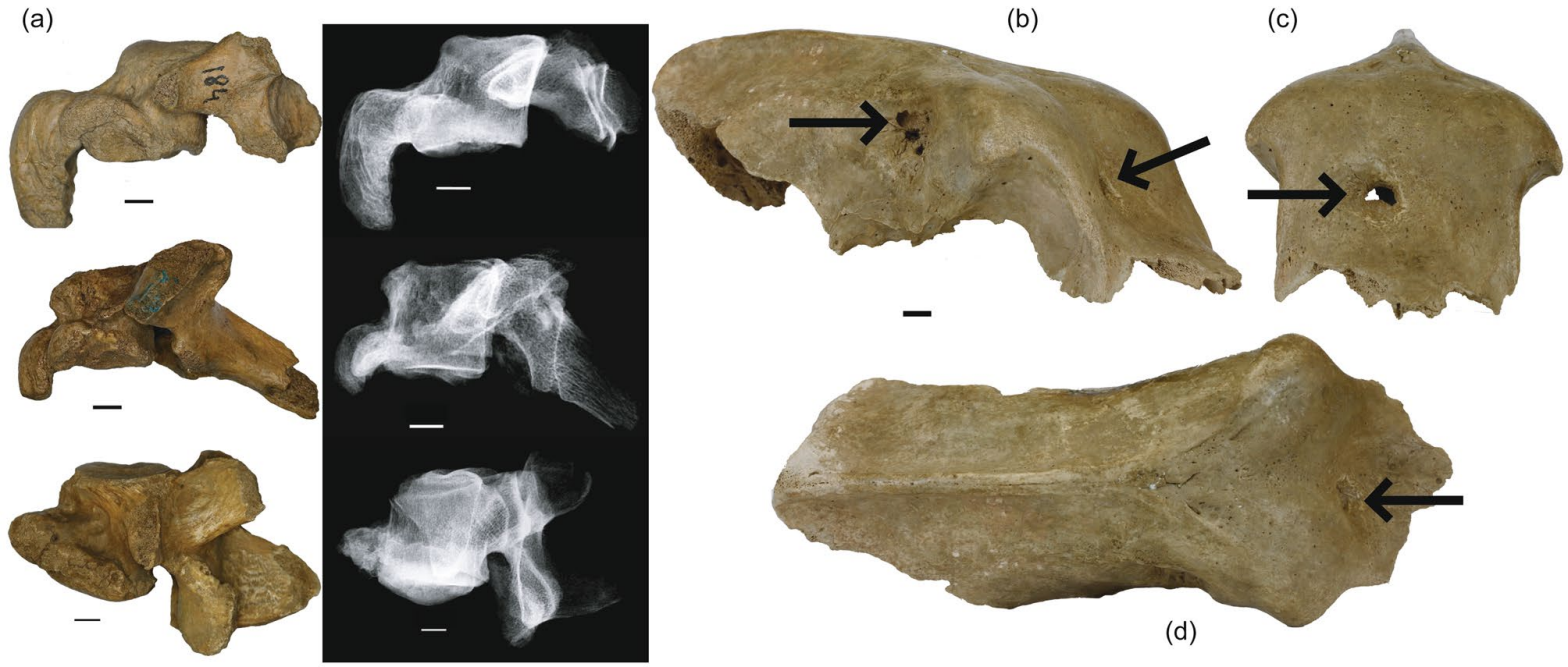
Palaeopathological modifications. (**a**) Left lateral views and X-ray images, of ossified *ligamenta flava anterior* in three *syndesmophytes*-affected vertebrae. These modifications are observed in various cervical, thoracic, and lumbar vertebrae of Niedźwiedzia Cave’s *Ursus spelaeus ingressus*. (**b**) Antero-right lateral view of the skull of a young female *Ursus spelaeus ingressus* from Niedźwiedzia Cave. The left arrow indicates an abscess, likely the direct cause of the individual's demise. The right arrow denotes a frontal bone perforation, likely resulting from the penetration of a tooth during an attack by another predator. (**c**) The same skull as shown in (**b**), presented in frontal view. The arrow indicates the frontal perforation shown in (**b**). (**d**) The same skull as in (**b**) and (**c**), displayed in dorsal view. The arrow indicates the same frontal perforation shown in (**b**) and (**c**). Scale bar 1 cm.

Pathological alterations were observed in over 21% of Niedźwiedzia bear vertebrae. Chronic diseases are revealed by bony outgrowths, joint alterations (arthrosis deformans), and fusion potential in advanced stages (Fig. 4a). Inflammation and degeneration are common, including spondyloarthrosis and ankylosing spondylitis. Tuberculosis and tumours are rare. Over 10% show degeneration, evenly in cervical, thoracic, and lumbar vertebrae. Notably, spondyloarthrosis was found in young individuals. This diversity in spine conditions, accurately assessed in this sample, underscores the range of pathological complexities within the Niedźwiedzia vertebrae. The extensively studied juvenile female bear skull from Niedźwiedzia Cave^28^ showes multiple pathologies: a frontal bone perforation indicating a healing process and a fatal abscess on the right parietal bone (Fig. 4b–d). Around 20% of the 2000 postcranial bones has Harris lines, and 3.1% displays transverse lines upon radiographic examination.

### Geochemical analysis

The silt deposits in each distinct profile share similar metal and biogenic element contents (Table 1). The analyses conducted on the sediments, along with the preliminary results of further in-depth analyses currently underway on the bones and teeth of the cave bears, have revealed particularly high concentrations of zinc.

**Table 1:** Average contents of pyrolysis losses (LOI), organic carbon (TOC) and other biogenic elements and selected metals in the mantle sediments of Niedźwiedzia Cave.

Profile	LOI	TOC	C	N	S	Na	K	Ca	Mg	Fe	Mn	Cu	Zn	Pb
	%					mg/g					μg/g			
JN-1	7.7	1.49	4.14	0.15	0.02	0.8	1.7	192	25.8	7.66	1718	72	1452	10
JN-2	6.8	1.26	3.92	0.13	0.02	1.14	1.69	182	24.6	6.94	1257	59	1391	12.3
JN-3	7	1.1	5.2	0.13	0.01	0.82	1.22	189	34.2	5.26	1548	54	1153	6.6

**Figure 5:**
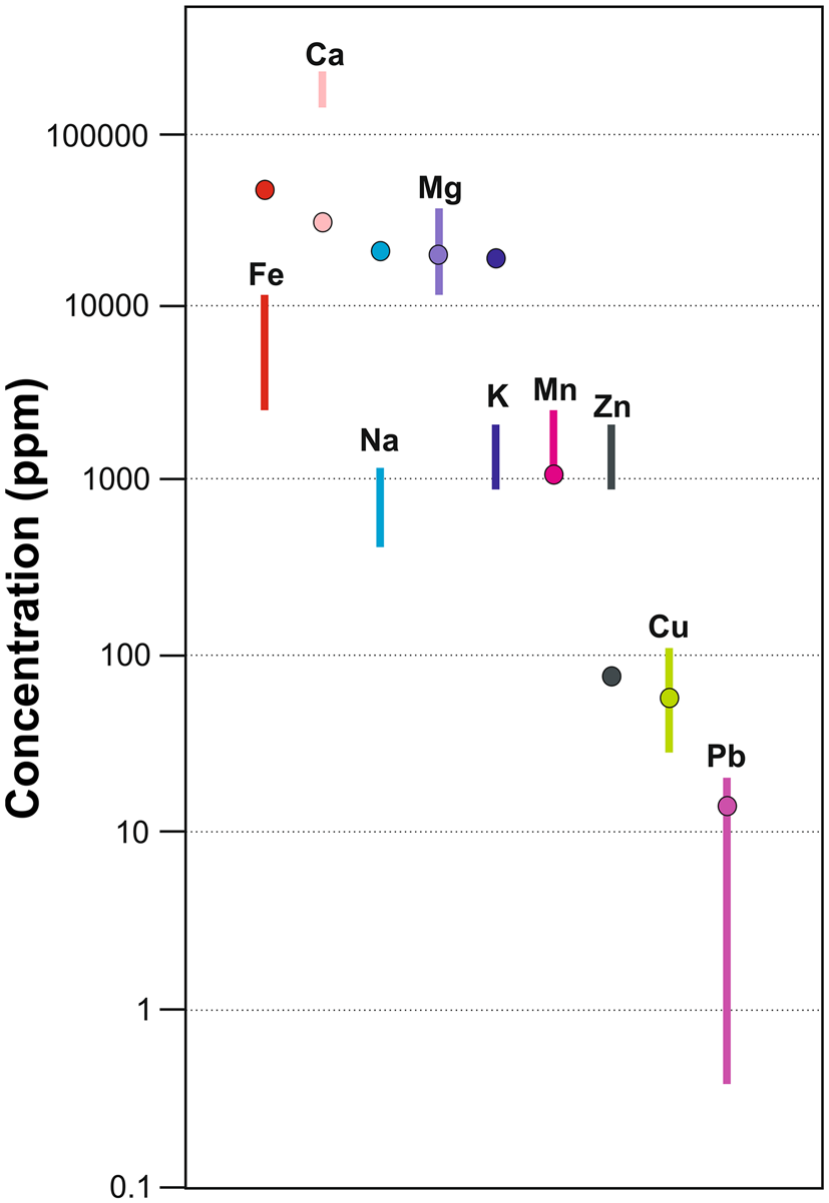
Sediment metal content in Niedźwiedzia Cave compared to Earth's crust. Lines indicate the cave's metal spectrum, circles the mean presence in Earth's crust^97^.

Zinc notably exceeds Earth’s crust levels, ranging from 1153 to 1452 μg/g (Fig. 5). Manganese and calcium levels are high, while iron, sodium, and potassium contents are lower than average (Fig. 5). Magnesium, copper, and lead hover around average crustal levels. Organic matter prevails in JN-1 sediments; JN-3 shows modest presence (Table 1, Supplementary Fig. S2). However, JN-3 reveals increased TOC and biogenic elements near the surface and at a depth of 50–80 cm (Supplementary Fig. S2). Phosphate concentration increases in upper layers of JN-2 and JN-3 (Supplementary Fig. S2). JN-1 displays diverse metal content compared to JN-2 and JN-3, likely due to redeposition (Supplementary Figs. S2 and S3). JN-3 water extracts show nitrite/nitrate disparities, higher at the top of the layer (Supplementary Fig. S4). Consistent chlorides and ammonium levels in the profile are noteworthy (Supplementary Fig. S4).

### Bone material and collagen extraction

Collagen from Niedźwiedzia Cave had a C/N ratio of 3.2–3.4, meeting established standards^29^. %C_coll_ was 34.9–43.0%, %N_coll_ was 12.0–15.3%, exceeding expected levels for well-preserved collagen^30^ (Supplementary Table S2). δ^13^C ranged from − 24.0 to − 20.0‰, δ^15^N from 1.1 to 7.2‰ (Supplementary Table S3). Niedźwiedzia Cave had a broader isotopic niche than Nietoperzowa, Perspektywiczna, and Medvedia Caves (Supplementary Fig. S5), a KUD value of about 19.95 vs. 6.56, 5.41, and 2.33 for Nietoperzowa, Perspektywiczna, and Medvedia Caves, respectively. At the chosen contour level (95%), the overlap between the isotopic niche spaces of Central European caves and Niedźwiedzia Cave ranged from 83 to 99%. Conversely, the isotopic niche space of Niedźwiedzia Cave exhibited an overlap of 12–30% with those from the Central European caves.

### Chronology

Results (Table 2) highlight a complex sedimentary history. JN-1 shows two date sets: the lower section, approximately 50 cm thick, yielded ages over 47,678 years BP, the upper, roughly 40 cm thick, encompassed dates ranging from 45,301 to 42,696 years BP. This stratigraphic pattern might be indicative of lithological influence. The lower section could likely contain redeposited sediments, whereas the upper may have accumulated in situ. JN-3 exhibits a narrower timeframe, around 49,000 years BP. A long bone excavated in the Primitive Man's Corridor was U-Th dated to 87,000 ± 6,000 years BP^15^. Further diverse dating analysis is crucial for a comprehensive understanding of the deposition of these layers.

**Table 2:** Radiocarbon dating of fossil remains obtained from the JN-1, JN-2, and JN-3 stratigraphic profiles in the intermediate level of Niedźwiedzia Cave.

Profile: date number	Laboratory number	Depth [cm]	^14^C date [yr BP]	Error [yr BP]	Calibrated ages [cal. yr BP] (2σ range—95.4%)
JN-1: 1	Poz-38572	40	40 800	900	45 301 (95.4%) 42 696
JN-1: 2	Poz-157019	40	42 000	2 000	52 322 (95.4%) 42 845
JN-1: 3	Poz-38569	50	50 000	3 000	… (95.4%) 48 525
JN-1: 4	Poz-157044	80	> 42 000		
JN-1: 5	Poz-38570	95	48 000	2 000	… (95.4%) 47 826
JN-1: 6	Poz-38567	95	47 000	2 000	… (95.4%) 46 836
JN-1: 7	Poz-38568	110	49 000	3 000	… (95.4%) 47 678
JN-1: 8	Poz-38565	110	> 49 000		
JN-1: 9	Poz-38566	110	> 51 000		
JN-1: 10	Poz-157045	125	> 42 000		
JN-2: 1	Poz-157047	40	> 46 000		
JN-3: 1	Poz-36468	ott-20	45 600	1 800	54 928 (5.6%) 53 547
					52 685 (89.9%) 45 294
JN-3: 2	Poz-36465	20–30	> 48 000		
JN-3: 3	Poz-157049	30	> 45 000		
JN-3: 4	Poz-36466	40–50	51 000	4 000	… (95.4%) 48 382
JN-3: 5	Poz-36467	50–60	> 50 000		
JN-3: 6	Poz-38564	60–70	50 000	3 000	… (95.4%) 48 525
JN-3: 7	Poz-38563	110–120	52 000	4 000	… (95.4%) 49 046
JN-3: 8	Poz-36469	130–140	48 000	2 500	… (95.4%) 47 264
JN-3: 9	Poz-38548	135–140	> 47 000		
JN-3: 10	Poz-38549	135–140	> 50 000		
JN-3: 11	Poz-38547	150	45 000	2 000	54 855 ( 4.8%) 53 584
					52 664 (90.6%) 44 668
	Poz-157050	170	40 000	2 000	49 740 (95.4%) 41 262
JN-3: 12	Poz-36470	170–180	45 000	1 600	54 382 ( 0.7%) 54 065
					52 484 (94.7%) 44 728
JN-3: 13	Poz-38550	185	43 800	1 600	51 770 (95.4%) 44 070
JN-3: 14	Poz-38546	190	> 51 000		

## Discussion

### Palaeobiological and taphonomic evidence

The balanced left-right ratio distribution of cave bear teeth indicates minimal disruption in material deposition or movement. The abundance of teeth and bones suggests regular *U. s. ingressus* hibernation at Niedźwiedzia Cave. The herbivorous reliance of these bears might have been challenged by shifts in primary producers, impacting local ecology and reflecting broader climatic dynamics^20^. Potential causes for the cave bears’ demise include (1) resource scarcity during hibernation, or (2) predation by carnivores, humans, or both. The former hypothesis presents avenues for additional explanations for the demise of these cave bears, including resource inaccessibility, unfavourable summers, and habitat fragmentation^31^.

Habitat fragmentation, likely due to ongoing glaciation, had cascading adverse effects on ecosystems^32–35^. Cave bears, highly specialised in a vegetarian diet, were particularly vulnerable due to their ecological limitations^13,14,23,26^. Their susceptibility stemmed from their strong dependence on the availability and composition of vegetation^20^. With sequential deterioration in environmental conditions and fragmentation of vegetation, younger bears faced demanding challenges in resource acquisition due to inexperience and competition, having a great impact on their survival during subsequent seasons. If the population decline relates to deteriorating conditions and vegetation fragmentation, younger bears likely suffered the most immediate consequences.

Cave bears vanished in the Sudeten Mountains during the late MIS 3 period, around 35–30 ka, not surviving the Last Glacial Maximum (LGM) from 23 to 19 ka^36^. Their extinction aligns with that of Europe’s megafauna, which started during Greenland interstadials 5–7 (GI-5 to GI-7), culminating at the onset of LGM^37^. Spelaeoid bear decline in Europe commenced between 50 and 45 ka^38^ and culminated during the coldest stadial, GS-3, spanning 27.5–23.5 ka^22^.

Climate cooling drove the extinction and migration of cave bears and most herbivores from the Sudetes before and during LGM^20,39^. Being strict herbivores, degradation in plant food quality due to climate change affected the bears’ diet^22,40^. Temperature decline likely disrupted their hibernation^22^, making them vulnerable to hunting^41,42^.

The significance of caves as hibernation sites increased competition with humans and other carnivores^22,43–45^. Niedźwiedzia Cave potentially served as a birthplace, fostering the formation of stable maternal groups for hibernation, similar to Spanish cave bears^46^. This link might have limited the finding of new hibernation sites, especially if occupied or frequented by competitors, like lions, hyenas, wolves, and humans. *Ursus arctos priscus*, a formidable carnivore, could also have played a role in supplanting and extinction of spelaeoid bears^8,47^. It was adapted better to continental climates than spelaeoid bears, and could dominate in competition for food and hibernation sites, especially in harsh periods. Brown bears infrequently used large caves, instead preferring hibernation near cave entrances, which potentially led to competition with cave bears^48^.

Taphonomic analysis at Niedźwiedzia Cave reveals evidence of trampling, such as networks of striations and linear grooves, and rare predator interactions, crucial for this study (Fig. 3). Occasional biting and gnawing marks found on bones suggest bear-carnivore encounters. It is unclear whether these are results of lethal attacks during hibernation, or scavenging of deceased bears, or of skeletal remains for minerals. In any case, these interactions suggest carnivores occasionally visiting bear carcasses, possibly being attracted by decomposition odours or accessing already skeletonised remains. The findings emphasise the complexity of carnivore interactions and their potential roles in scavenging and mineral acquisition from bear remains in the cave.

No evidence supports human hunting on cave bears in Niedźwiedzia Cave. Neither conclusive human presence nor signs of the cave hyena *Crocuta crocuta spelaea* are present, with no reliable records of this species in the Sudeten region^8,10^. Diedrich^43^ suggests hyenas specialised on cave bears in European boreal mountain forests, possibly due to higher cave bear densities and a lack of mammoths and steppe megafauna. However, Sudeten caves, with scarce ungulate records and ample bear lairs, lack evidence of this hyena.

Cranial bone corrosion, not seen in teeth, indicates highly alkaline cave conditions that impede bone dissolution. Stable, moisture-saturated, alkaline environments prevent bone dissolution by saturating pore water with Ca^2+^ and [PO_4_]^3−^ ions. Conversely, wet-dry cycles expedite dissolution^49^.

Manganese staining on the bear remains in Niedźwiedzia Cave confirms alkaline conditions, linked to bacterial activity in moist, mildly alkaline environments^50^. McAdams et al.^51^ suggest that guano-induced microorganisms generate sulphur compounds in moist, anoxic settings, fostering an alkaline environment. These findings, along with the ascertained taphonomic modifications, suggest that the bear remains likely encountered bacterial colonies thriving in anoxic guano deposits amid wet-dry conditions.

Age-scoring and survival analyses for Niedźwiedzia cave bears show a peak mortality rate (27%) between 1.5 and 3 years (stage II), and 17% for newborns (< 1.5 years) and 11% for individuals aged 12–16 years. This aligns with Schwabenreith-Höhle’s findings, emphasising stage II prevalence^52^. Unlike Slovenian (DivJe babe I, Mokrica, and Potocka zijalka), Belgian (Goyet), Romanian (Peștera cu Oase), and German (Bärenhöhle) sites with abundant stage I remains, Niedźwiedzia reveals different mortality patterns^53–58^.

The mortality profile of Niedźwiedzia cave bears, classified as juvenile, prime, and old adults, aligns with the non-violent attrition pattern often linked to hibernation deaths^59^. New-born bear mortality might stem from resource scarcity during prolonged hibernation, potentially exposing them to predator risks. Older bears face mortality risks due to food scarcity, predator encounters, and mating competition. Fluctuating mortality rates suggest environmental impact on survival. Limited resources could signal individual incapacity or broader environmental depletion, influencing species decline or reflecting climate changes in their habitat. The latter hypothesis provides insight into the environmental trajectory affecting these late-stage cave bears.

### Palaeopathology

Palaeopathology, through the study of fossilised remains^60^, reveals disease causes and progression^61^. Bone alterations uncover conditions affecting structure, like inflammation and necrosis^62^. Chronic diseases leave distinct marks in bones, offering insights into past animal health and ancient environments.

Forelimb fractures in *U. s. ingressus* at Niedźwiedzia Cave suggest increased risk compared to hind limbs, likely due to behaviours involving digging, hunting, or conspecific confrontations. Potential tuberculosis instances in the population point to this ailment’s presence. This is significant epidemiologically, questioning its transmission and impact on the health of the ancient bear population. Tuberculosis typically originates from primary foci, spreading metastatically. Bacteria causing tuberculosis exhibit osteolytic activity under favourable conditions, but their precise role in pathologies of the skeletal system remains unclear^63^.

The abscesses found in the remains of the Niedźwiedzia bear imply exposure to risks of physical injuries, likely resulting from conflicts, such as encounters with carnivores or similar species. This insight highlights environmental challenges they faced. Severe periosteal inflammation leads to bone resorption, as seen in the radius bone, indicating advanced inflammation consequences with necrotic regions developing around irritants^63^.

Degenerative spinal issues, constituting 50% of the Śnieżnik Pleistocene bear population’s ailments, were predominant. Factors like species' weaknesses, climate, and cave habitation might have influenced these conditions. Rare rheumatoid-like conditions found in Niedźwiedzia bears could have hindered mobility and defence, possibly contributing to some cases of death. Spinal joint degeneration, irrespective of age, likely impacted the bears’ viability, limiting mobility and affecting foraging and defence abilities. This suggests that these spinal diseases significantly influenced the bears’ survival and behavioural capabilities in the Pleistocene environment.

The prevalence of rickets in these Pleistocene bears might stem from unidentified environmental factors of the Śnieżnik Massif, affecting immunity and susceptibility to infections. Pathological alterations in the juvenile bear skull, resembling marks from sharp tools, potentially hint at human interaction, challenging presumed limited Palaeolithic human-bear contact. These cranial injuries, resembling those observed in criminological and archaeological contexts^62,64^, may erroneously suggest human involvement in these ancient bear injuries. Nonetheless, these injuries were, in fact, caused by carnivores, challenging prevailing beliefs about Palaeolithic human-bear interactions.

The frontal bone damage in the juvenile female bear, initially thought to be human-inflicted, was re-interpreted as a bite mark from a carnivorous predator based on injury morphology^65^. The bear survived the initial blow but succumbed to an illness months later^28^. Healing indicated survival, followed by death due to an abscess extending to the meninges. The carnivore’s identity—another cave bear, a steppe brown bear (paralleling modern bear behaviours^66^,^67^), or the cave lion—remains uncertain. However, the extent and marks of the skull’s damage aligned with lion canines suggest lion interaction and possibly human involvement, raising alternative hypotheses^8,28^.

In Siberian tiger confrontations with brown bears, the tiger leaps, holds the bear’s head, and bites its cervical vertebrae^68^. Similar scars on the cave bear’s skull suggest an encounter with *Panthera s. spelaea* demonstrating similar behaviour^8,48,68^. Comparable skull injuries from carnivore activity are seen in remains from European caves^43,48,69^. Niedźwiedzia Cave yielded remains of at least ten individuals of *P. s. spelaea*—nine males and a lioness^8,70^. Robust males hint at specialised hunts in Sudeten caves^71^. Lions, resembling robust *P. atrox*, with powerful forelimbs, likely hunted cave bears within narrow caves^72^. Food scarcity drove Pleistocene lions into caves to hunt on bears^8,43,48,70^. Lions risked confronting bears in darkness but subdued prey with fatal bites, often succumbing in encounters. Non-scavenged lion carcasses accumulated in caves, explaining unusual bone deposits^43,48^.

The histological analysis suggests that Sudeten cave bears occasionally faced malnutrition during autumn, akin to seasonal dietary patterns of brown bears. These occurrences, possibly linked to food availability fluctuations, notably affected bears aged 1–4 years. Despite these intermittent periods of famine, they had limited impact on the developmental trajectories of Pleistocene bears.

### Geochemical analysis

The multivariate correlation analysis (Supplementary Fig. S6) delineates significant associations among metals and biogenic elements within the silt samples. Strong positive correlations (r > 0.9) were observed between K and Fe, as well as Zn and Fe. Additionally, moderate positive correlations (r = 0.70–0.89) were noted between TOC, biogenic elements (N, S), K, Fe, heavy metals (Zn, Cu, and Fe), and sulphur (Supplementary Fig. S6). These findings suggest that heavy metal occurrence in cave muds may be linked to iron oxides, hydroxides, and the clay fraction, particularly indicated by potassium concentrations. Furthermore, the correlation of certain heavy metals (e.g. Cu, Zn, and Fe) with sulphur implies their potential presence in sulphide form in the silt.

In alluvial sediments of the Niedźwiedzia Cave, heavy metal sulphides likely stem from mineralisation at the limestone-gneiss interface^73^. Ore minerals—sphalerite (ZnS), chalcopyrite (CuFeS_2_), pyrite (FeS_2_), arsenopyrite (FeAsS), pyrrhotite (FeS), and covelline (CuS), among others—are implicated^73^. Weathering zones may transport heavy metals, possibly linked to clay minerals and iron compounds, into the cave mud^73^. Plants, particularly birch and willow species thriving in zinc-rich soils, might accumulate a significant amount of zinc^74^. Despite zinc-rich ores in the Śnieżnik Massif, pinpointing the source of excess zinc in cave silt remains elusive. Interplenivistulian conditions, fostering birch and willow growth, potentially fuelled zinc accumulation^74,75^. LA-ICP-MS analysis of cave stalagmites showed varying zinc levels in drip waters^76^. Zinc concentrations peaked sporadically at 150–180 ppm during the 3.0–0.3 ka period^76^.

The herbivorous cave bears likely fed on zinc-rich birch and willow twigs in the Śnieżnik Massif, possibly accumulating zinc, especially in mineralised zones. This zinc might have entered Niedźwiedzia Cave via soil solutions carrying zinc-enriched clay-organic mixtures from both biogenic and geogenic sources. Biogenic enrichment of cave silt by faeces and decomposed animal remains, particularly cave bears, might have also contributed. A comprehensive understanding of the high zinc levels in the cave’s silt warrants further research, particularly examining zinc levels in rocks, weathered materials, soils, specific plants from the Kleśnica and Kamienica catchment areas, and in cave bear bones and teeth. Presently, ongoing analyses focus on the zinc content in bear bones.

### Stable isotope analyses

The isotopic analysis of cave bears from Niedźwiedzia Cave suggests a herbivorous diet, but their significant isotopic variability, differing from other sites, requires thorough scrutiny^77^. The divergence in δ^13^C values may arise from the sample’s chronology, possibly indicating older periods for Niedźwiedzia Cave bears, shaping distinct ecosystems. The wide-ranging δ^15^N values might imply diverse dietary components or physiological factors. Some researchers^78,79^ link δ^15^N variability to an omnivorous diet, yet definitive proof remains elusive due to the absence of isotope data from coeval species at the site. Additionally, pathological bone alterations might contribute to δ^15^N variability, despite efforts to exclude visibly affected samples. Intraskeletal variations in isotopic compositions in pathological individuals could reach up to 2.5‰ for δ^15^N. However, the influence of this signal on palaeodietary interpretations relies on the type of ailment, bone sampling area, and individual diet^80^. Overall, the substantial isotopic niche breadth among cave bears of Niedźwiedzia Cave, compared to other caves sites in Central Europe, likely arises from two key factors: chronological differences and the presence of pathologically altered bones. These findings emphasise the need for further research, especially involving isotope data of coeval species and a detailed analysis of pathological alterations in bones, in order to solidify interpretations of cave bear diets and the factors influencing their isotopic variability^24,77–79,81^.

## Conclusions

The comprehensive analysis of bone remains from Niedźwiedzia Cave unravels critical aspects of the ecology of the Pleistocene cave bear *U. s. ingressus*. It provides insights into their hibernation patterns, reflecting minimal disturbance across excavated levels. The possible extinction scenarios suggest an array of challenges—hibernation-linked mortality, post-hibernation resource scarcity, and shifts in climatic conditions during glaciation. While the evidence suggests limited human involvement and minor interspecific predation, broad-scale European factors, including cooling climates and competition with *U. a. priscus*, likely influenced their extinction. Due to its high specialization in a herbivorous diet, we can assume that a primary factor contributing to its extinction was a decline in the availability of plant food associated with climate cooling.

Histological analyses reveal sporadic malnutrition episodes, notably affecting young bears due to seasonal food variations, highlighting environmental impacts on resource availability^82^. Pathological findings, from forelimb fractures to severe infectious diseases such as tuberculosis, abscesses, and rickets, underscore the vulnerability and health challenges of bears in the cave environment. The studied cave bear was prone to injuries and bone fractures, for example, during activities such as searching for food or engaging in confrontations with other individuals.

The remains of the cave bear in Niedźwiedzia Cave were preserved from dissolution due to highly alkaline conditions after saturation with calcium and phosphate ions. Following deposition, the remains were colonized by bacteria thriving in anoxic guano deposits. The enigmatic anomaly of high zinc concentrations in cave silt raises intriguing questions about its source. This could be linked to the assimilation of zinc from herbivorous diets or accumulated bones of generations of bears that used the cave, potentially influencing their health and contributing to their decline due to the harmful effects of excess zinc. Further studies on zinc concentration in both the bones and the surrounding region of the cave are necessary to address this issue.

The discoveries from Niedźwiedzia Cave provide valuable insights into the complex interplay of environmental conditions, dietary behaviour, and stressors influencing Pleistocene cave bear lives. They demonstrate a nuanced picture of environmental deterioration as a possible contributor to the species’ gradual extinction in the Śnieżnik Massif region during the Pleistocene.

## Materials and methods

### Palaeobiology and taphonomy

The study analysed 2122 cave bear cheek teeth from Niedźwiedzia Cave across seven areas, merging data from three nearby excavation sites for statistical analysis. Six areas underwent detailed examination following a comprehensive protocol (Fig. 1; see Supplementary SI-1 and Supplementary Table S1). The analysis involved:

- Anatomical and taxonomic identification, using statistical tests to detect biases and categorise teeth by developmental stages and distribution patterns.
- Employing the minimum number of individuals (MNI) for individual categorisation^83^.
- Identifying bone damage caused by different factors, distinguishing between carnivore and human-induced alterations^84^.
- Utilising Stiner’s^59^ age-scoring technique to determine the demographic structure of the bear assemblage. Using tooth eruption and wear, this technique identifies nine developmental stages that approximate true ages, resistant to damage and forming crucial foundational data for analysis^59,85^.
- Analysing mortality patterns through wear stages, survivor counts, age-specific mortality rates, and survival time curves across cave levels and areas.

The study combined tooth-based ontogenetic ages with postcranial estimates and employed various statistical analyses and software packages while controlling false discovery rates to ensure reliable results with a significance level below p < 0.05.

### Palaeopathology

The detailed analysis of approximately 2000 of *U. s. ingressus* bones from Niedźwiedzia Cave, housed at the University of Wrocław (Poland), employed diverse methods (see Supplementary SI-1). This extensive study aimed to uncover an array of pathological changes shedding light on the bears’ adaptations to harsh environmental conditions. Additionally, it aids in comprehending the potential factors contributing to the eventual extinction of these bears in the Late Pleistocene^82,86^.

### Geochemical analysis

Samples from different silt profiles in Marten’s and Primitive Man’s Corridors underwent thorough geochemical analysis, revealing specific sediment characteristics and elements present (Fig. 1, see Supplementary SI-1 and Supplementary S1). These analyses involved assessing organic matter, carbon, nitrogen, sulphur, and phosphate content, as well as concentrations of various elements through specialised laboratory techniques.

### Fossil material and collagen extraction

New isotopic analysis of cave bears from Niedźwiedzia Cave was compared with existing Central European data (see Supplementary SI-1 and Supplementary Tables S2 and S3). The study examined 39 adult remains—31 bones and 8 teeth—using collagen to understand their diet and tooth development, e.g.^30,87^. Altitude-adjusted isotopic data from other caves were considered for comparison^24^. Isotopic data underwent comparison using isotopic niche space analysis and the Kernel Utilisation Density (KUD) model, calculated at a 95% contour level with the rKIN package in R software^88,89^. Collagen extraction involved a modified Longin method with NaOH treatment, followed by carbon and nitrogen measurements^30^. Analysis at the University of Warsaw ensured precision below 0.1‰ for δ^15^N and below 0.2‰ for δ^13^C^90^.

### Chronology

Chronological analysis of *U. s. ingressus* bones from Niedźwiedzia Cave involved radiocarbon and U–Th dating (see Supplementary SI-1). Radiocarbon dating used collagen extracted from bones^91,92^. U–Th dating at the U–series Laboratory in Warsaw included cleaning^93^, collagen assessment^30^, and alpha-spectrometric measurements to compute ages based on activity ratios, adjusting for initial thorium contamination^94,95^.

### Supplementary Information

Supplementary Figure S1.

Supplementary Figure S2.

Supplementary Figure S3.

Supplementary Figure S4.

Supplementary Figure S5.

Supplementary Figure S6.

Supplementary Information 7.

Supplementary Table S1.

Supplementary Table S2.

Supplementary Table S3.

## Data Availability

All data generated or analyzed during this study are included in this published article and its Supplementary Information file.
