# Supplementary figures and images for: Fate and preservation of the Late Pleistocene cave bears from Niedźwiedzia Cave in Poland, through taphonomy, pathology, and geochemistry
Source: Sci Rep. 2024 Apr 29;14:9775. doi: 10.1038/s41598-024-60222-3 (PMC11059340; doi:10.1038/s41598-024-60222-3)

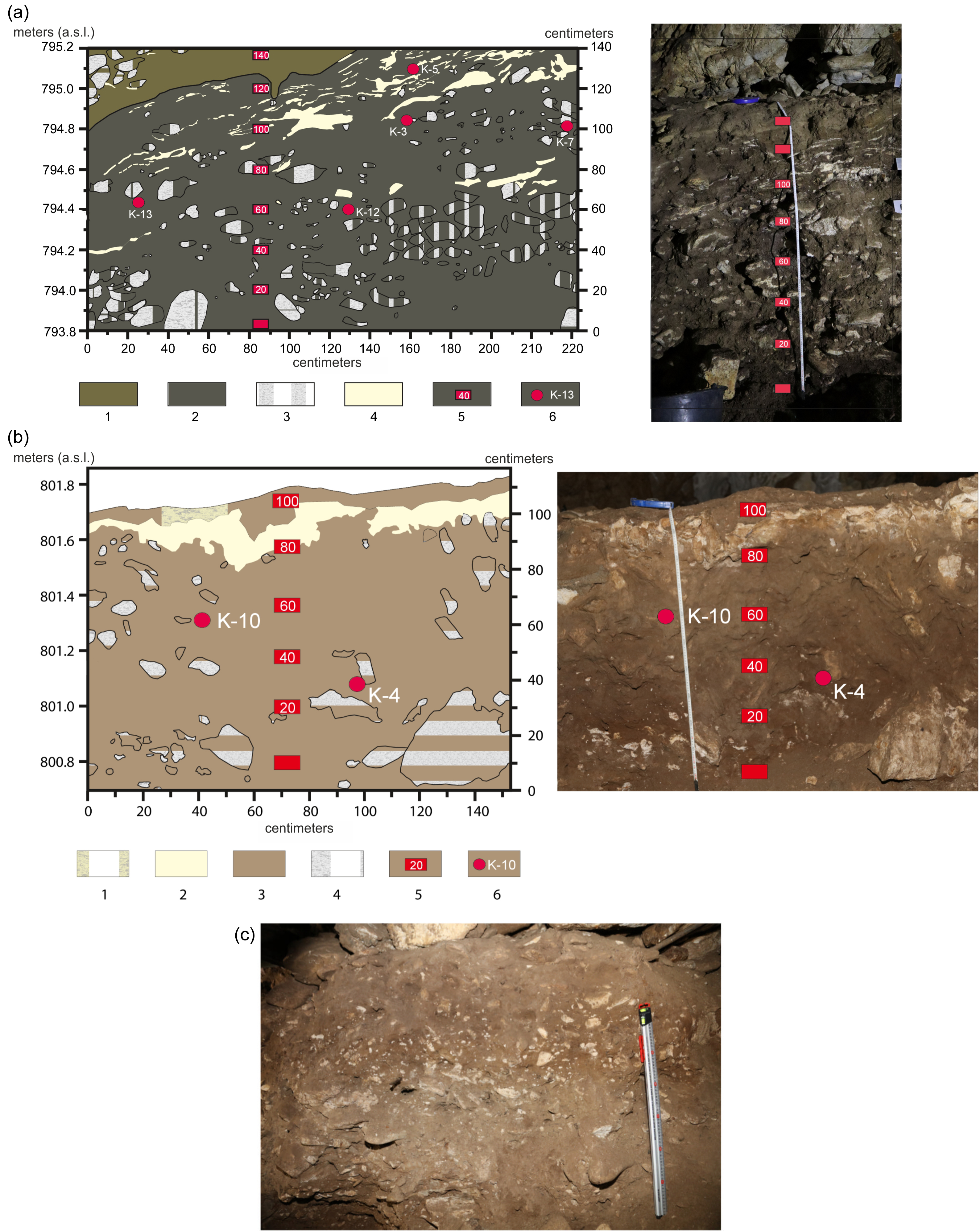

Supplement: Supplementary file 1 — Supplementary Figure S1. [file 41598_2024_60222_MOESM1_ESM.jpg]

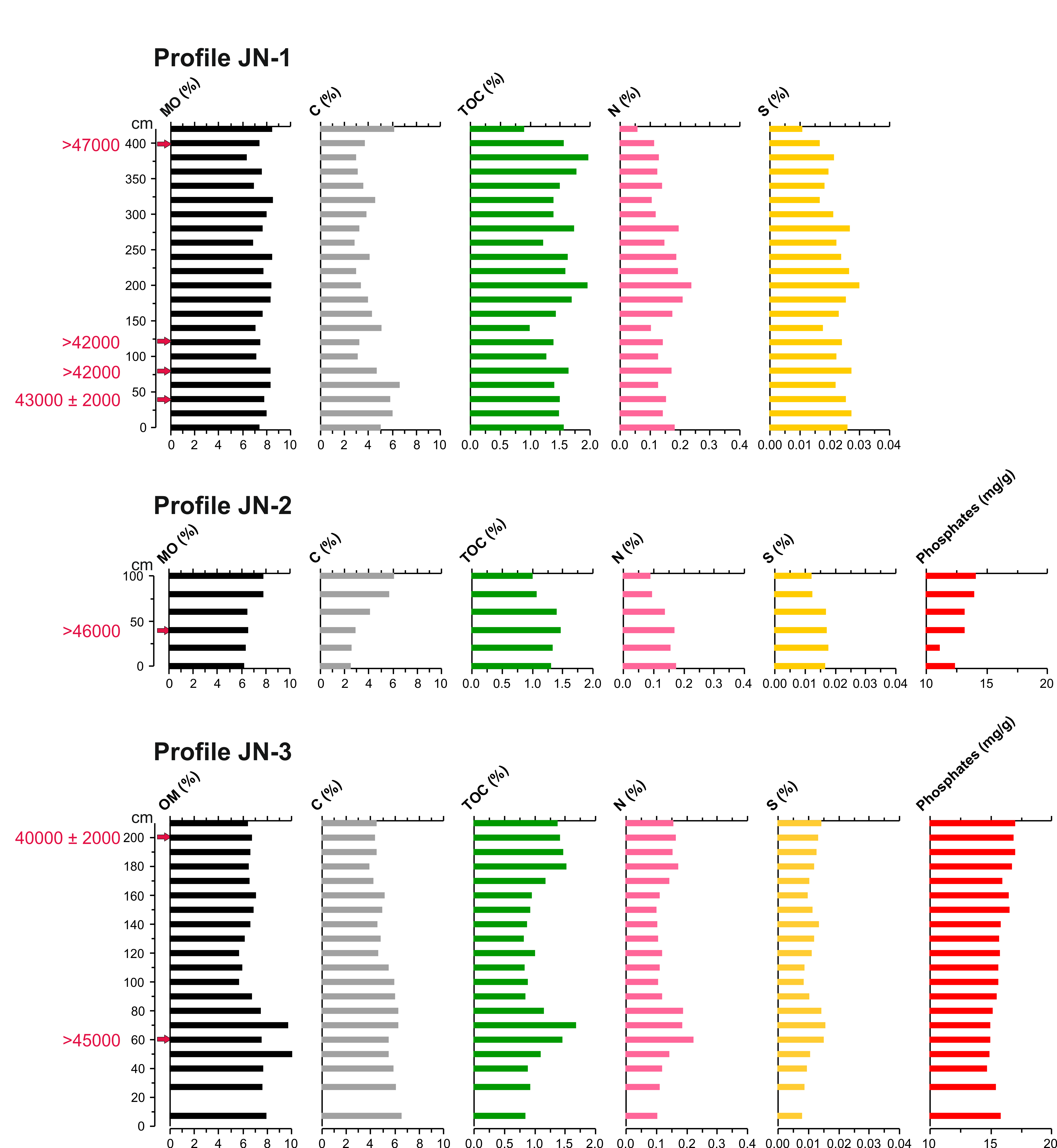

Supplement: Supplementary file 2 — Supplementary Figure S2. [file 41598_2024_60222_MOESM2_ESM.tif]

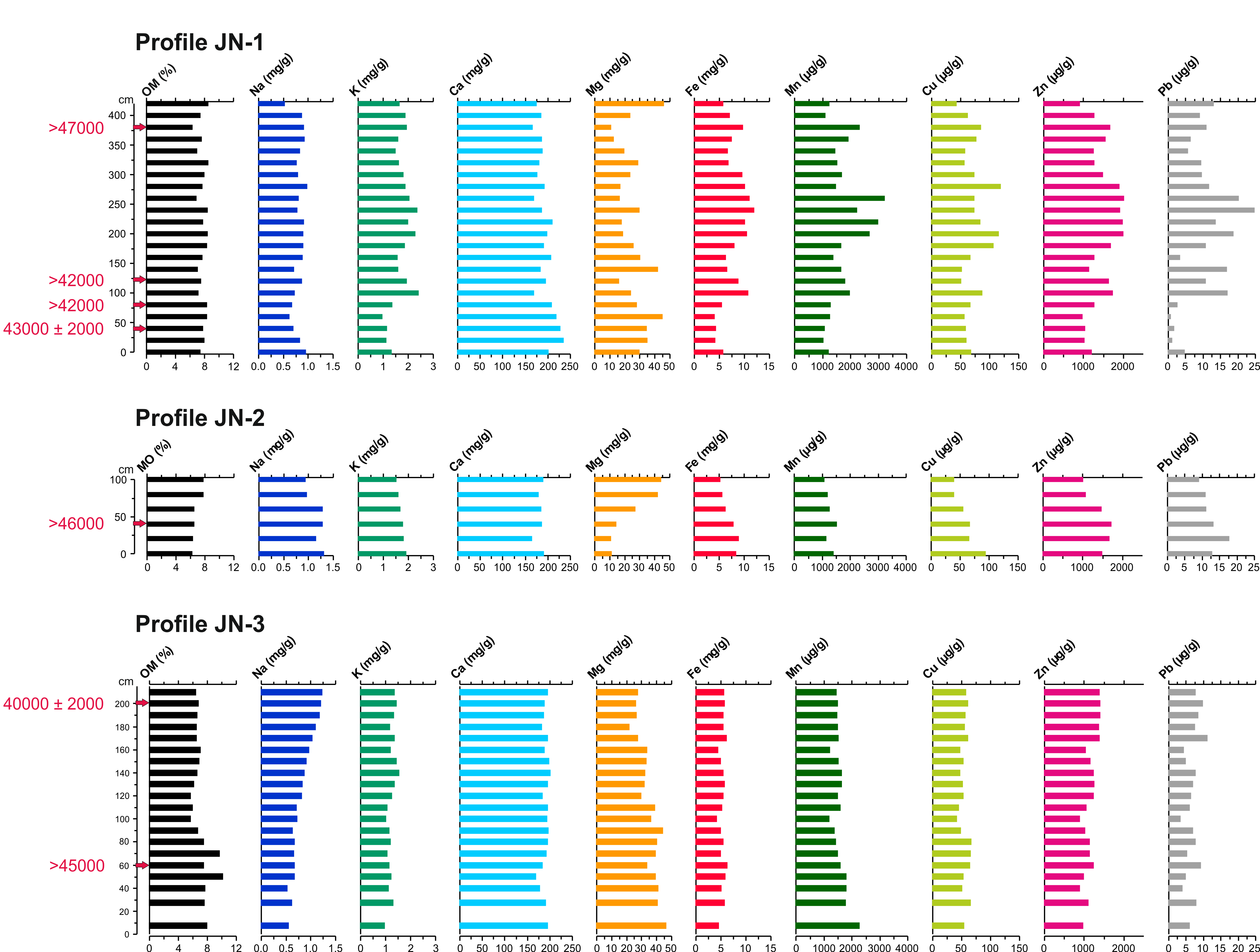

Supplement: Supplementary file 3 — Supplementary Figure S3. [file 41598_2024_60222_MOESM3_ESM.tif]

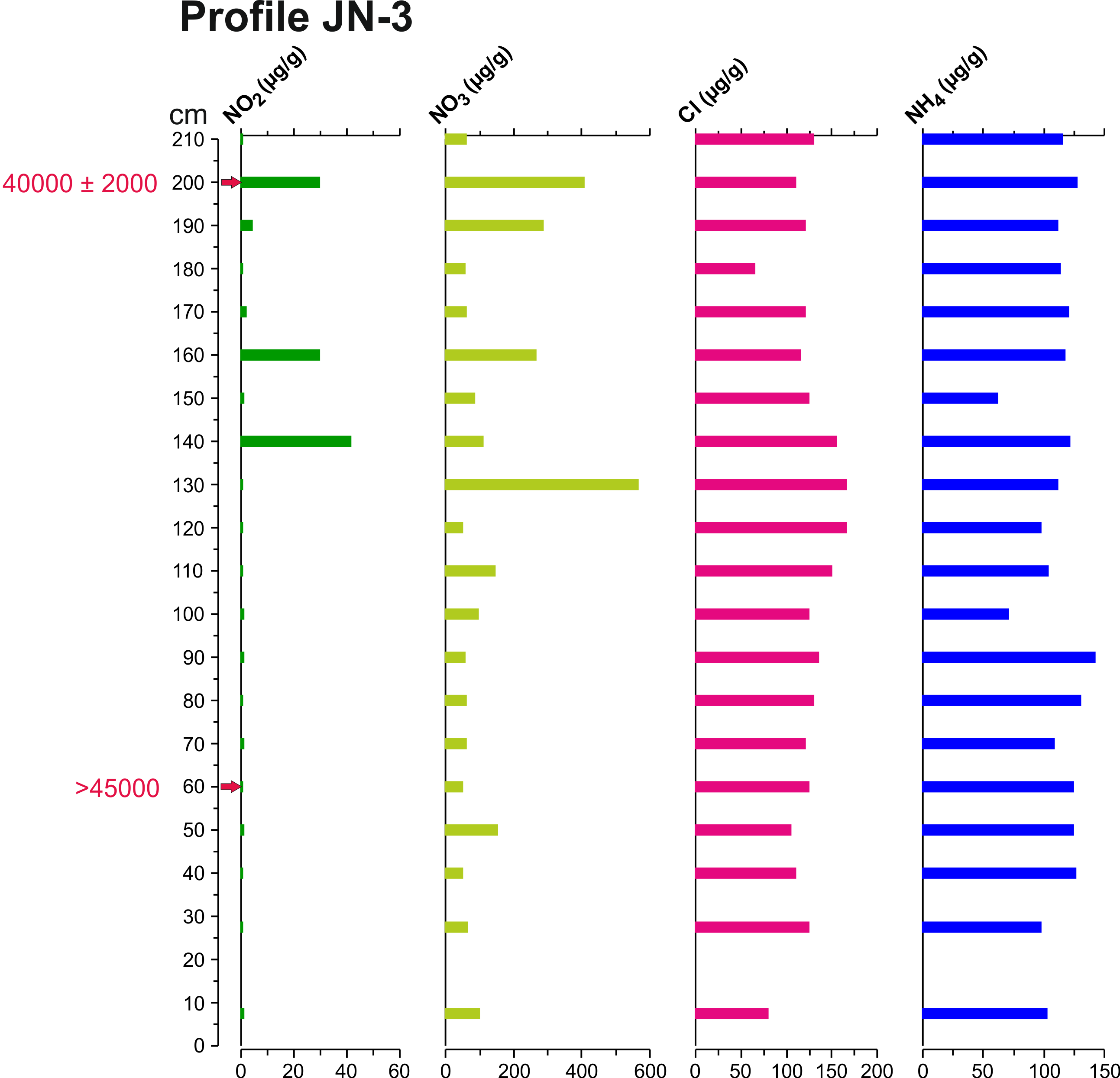

Supplement: Supplementary file 4 — Supplementary Figure S4. [file 41598_2024_60222_MOESM4_ESM.tif]

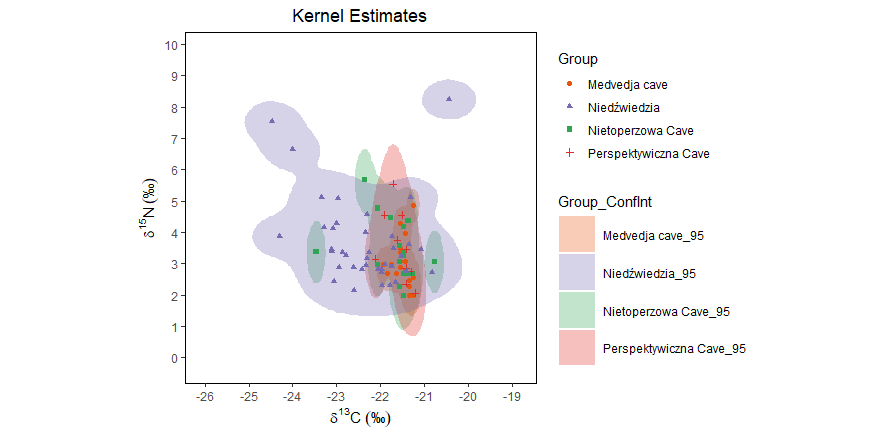

Supplement: Supplementary file 5 — Supplementary Figure S5. [file 41598_2024_60222_MOESM5_ESM.png]

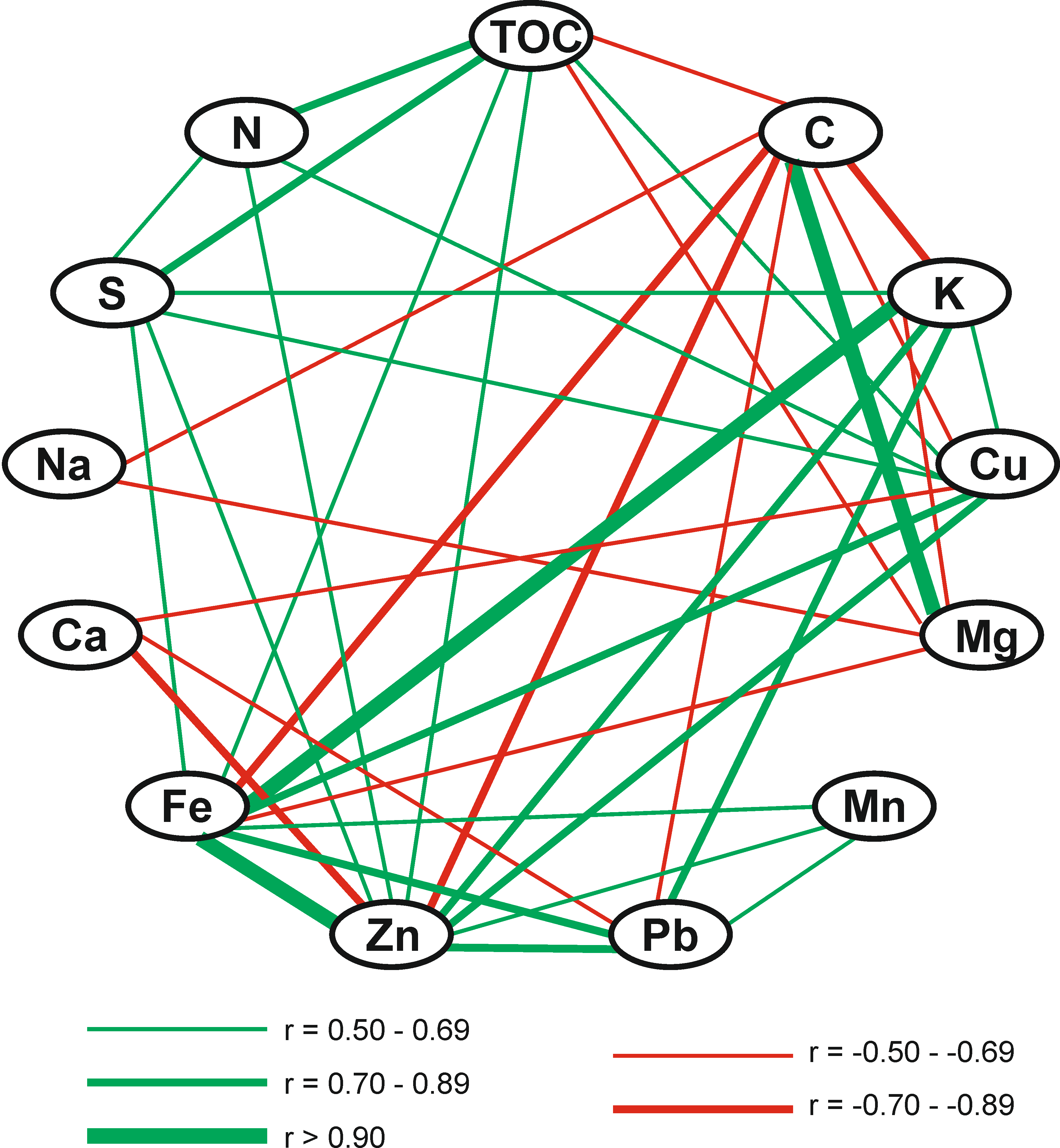

Supplement: Supplementary file 6 — Supplementary Figure S6. [file 41598_2024_60222_MOESM6_ESM.tif]
