# Supplementary material for: Fate and preservation of the Late Pleistocene cave bears from Niedźwiedzia Cave in Poland, through taphonomy, pathology, and geochemistry
Source: Sci Rep. 2024 Apr 29;14:9775. doi: 10.1038/s41598-024-60222-3 (PMC11059340; doi:10.1038/s41598-024-60222-3)
Supplement: Supplementary file 7 — Supplementary Information 7. [file 41598_2024_60222_MOESM7_ESM.docx]

**Supplementary information**

**Fate and preservation of the Late Pleistocene cave bears from** **Niedźwiedzia Cave in Poland, through taphonomy, pathology, and geochemistry**

Adrian Marciszak^1^, Paweł Mackiewicz^2^, Ryszard K. Borówka^3^, Chiara Capalbo^4^, Piotr Chibowski^5^, Michał Gąsiorowski^6^, Helena Hercman^6^, Bernard Cedro^7^, Aleksandra Kropczyk^1^, Wiktoria Gornig^8^, Piotr Moska^9^, Dariusz Nowakowski^10^, Urszula Ratajczak-Skrzatek^1^, Artur Sobczyk^11^, Maciej Sykut^12,13,14^, Katarzyna Zarzecka-Szubińska^1^, Oleksandr Kovalchuk^1,15^, Zoltán Barkaszi^15,16^, Krzysztof Stefaniak^1^, Paul P.A. Mazza^4*^

^1^ University of Wrocław, Department of Palaeozoology, Wrocław, Poland

^2^ University of Wrocław, Department of Bioinformatics and Genomics, Wrocław, Poland

^3^ Szczecin University, Institute of Marine and Environmental Sciences, Szczecin, Poland

^4^ University of Florence, Department of Earth Sciences, Florence, Italy

^5^ Faculty of Biology, Biological and Chemical Research Centre, University of Warsaw, Warsaw, Poland

^6^ Polish Academy of Sciences, Institute of Geological Sciences, Warsaw, Poland

^7^  University of Szczecin, Institute of Marine and Environmental Sciences ^8^ University of Wrocław, Department of Evolutionary Biology and Conservation of Vertebrates, Wrocław, Poland

^9^ Silesian University of Technology, Institute of Physics – Centre for Science and Education, Gliwice, Poland

^10^Wrocław University of Environmental and Life Sciences, Division of
Anthropology, Wrocław, Poland

^11^ University of Wrocław, Institute of Geological Sciences, Wrocław, Poland

^12^ Center for Ecological Dynamics in a Novel Biosphere (ECONOVO), Department of Biology, Aarhus University, 8000 Aarhus C, Denmark

^13^ Department of Archaeology and Heritage Studies, Aarhus University, Moesgård Allé 20, DK-8270 Højbjerg, Denmark

^14^ Mammal Research Institute, Polish Academy of Sciences, Stoczek 1c, 17-230 Białowieża, Poland

^15^ National Academy of Sciences of Ukraine, National Museum of Natural History, Kyiv, Ukraine

^16^ John von Neumann University, Department of Agricultural Sciences, Kecskemét, Hungary

*Corresponding author

**SI-1**

**Material and methods**

Palaeobiology and taphonomy

1. The distribution of left and right upper and lower cheek teeth was compared to an expected 1:1 ratio to identify potential preservation or mixing issues using Fisher's exact test. Selected levels underwent further analysis, categorizing teeth into nine developmental stages^1^. Statistical tests including G-test, Pearson's Chi-squared test, Kolmogorov-Smirnov test, and Spearman’s rank correlation assessed distributions, aiming to identify potential correlations in tooth distributions across levels.

2. MNIs were calculated considering dentition and limb bones. Limb bone MNI was assessed by side-matching based on size, proportions, and preservation^2^. Age estimates and bone symmetry aided in MNI calculations. Cheek teeth analysis compared to limb bone MNI for accuracy. Contextual factors did not affect MNI due to distinct depositional units. The analysis of the entire tooth sample reduced the impact of specimen aggregation on MNI counts in the cave bear sample.

3. The analysis of pre- and post-depositional alterations encompassed a spectrum of taphonomic phenomena, such as trampling, potential root etching, corrosion of diverse nature, and abrasion/polishing. The careful scrutiny of bone surface damage formed a pivotal aspect of probing interactions among Niedźwiedzia bears, other carnivores, and human agents. This scrutiny entailed the discernment of bites, punctures, gnaw marks, and possible tool-induced alterations. It was of paramount importance to distinguish gnaw marks originating from carnivores, characterized by pitting, deep U-shaped grooves, irregular festooned edges, and concentration on bone ends, from those intentionally introduced by human agency. There is an inclination to encompass a broad spectrum of incisions in the category of cut marks. In the present study, a criterion was adopted that adhered to a precisely outlined set of well-defined characteristics. These marks are usually straight, deep, insisted, parallel incisions with narrow cross-section characterized by either symmetrical or asymmetrical walls. They are predominantly concentrated in specific skeletal regions, especially targeting anatomical segments of nutritional significance in terms of meat, fat, and/or marrow. They cluster around joints, or on diaphyseal surfaces, responding to systematic logics governing carcass processing and butchering. These regions supposedly remain shielded from incidental trampling scratches and grooves. Recognizing these distinctions sheds light on possible specific carcass processing techniques, such as skinning, filleting, disarticulation, and fracturing.

4. The age-scoring method adopted in this study^1^ determined bear assemblage demographics and analysed generational mortality patterns via cheek teeth, skulls, and postcranial specimens. This technique, based on tooth eruption and wear, identifies nine developmental stages approximating true ages (Table **S1**), resilient to biostratinomic damage, providing foundational data for analysis.

5. MNI distributions based on wear stages (ages) calculated individuals dying in intervals (D) across levels and areas, compared with the global MNI. Statistical analysis employed G-test, Pearson's Chi-squared test, Kolmogorov-Smirnov test, and Spearman’s rank correlation coefficient. Survivor counts (S) were calculated using the following formulas:

$$S_{1}=\sum_{i=1}^{9} D_{i}$$

and for *i* $\in\left\langle2;9 \right\rangle$: $S_{i}=S_{i-1}-D_{i-1}$,

where *i* is the stage of tooth wear class (age).

Given the premise, we calculated the age-specific mortality rate (R), representing the proportion of individuals surviving at the start of an interval who pass away within that specific interval:

$R=D_{i}/S_{i}$ for *i* $\in\left\langle1;9 \right\rangle$

The Kaplan-Meier method estimated survival times and probabilities, presented through survival time curves. Comparisons of MNI-derived survival curves across cave levels and areas were made against a global MNI curve. Log-rank (Mantel-Haenszel), Peto & Peto modification of Gehan-Wilcoxon, and Mantel-Cox tests were employed. The age-scoring protocol by Stiner ^1^ clusters the eruption-wear stages into three broader age categories aligning with physiological and ethological changes in female mammals: juveniles (I-III), prime adults (IV-VII), and old adults (VIII-IX).

The postcranial estimates, complementing the tooth-based ontogenetic ages, considered size, porosity, bone development, muscle attachments, cancellous bone, and epiphyseal fusion^3^. While less precise than tooth-based assessments, these estimates categorized specimens as infants (cancellous bones), juveniles (partially unfused epiphyses), and adults (fully fused epiphyses). Statistical analysis relied on R software^4^, utilizing packages like stats, DescTools, biostatUZH, ggsurvfit, and survival. To address multiple hypotheses, the Benjamini-Hochberg method controlled false discovery rates, setting significance below a p-value of 0.05 for reliable results.

Palaeopathology

The examined skeletal materials included cranial and postcranial elements like long bones, flat bones, and vertebrae. Following systematic research protocols, morphological, radiological, and histological techniques were employed to comprehensively examine bone tissue. This approach identified pathological changes, determined ontogenetic age, examined Harris lines, and assessed bone tissue loss macroscopically and microscopically. The lines, indicating bone density changes and stress, drew attention to health and dietary concerns. These transverse lines provided insights into ontogenetic and population dynamics, enriching understanding of how organisms respond to adverse environments and contributing to unravelling factors behind the Śnieżnik Massif *U. s. ingressus* extinction in the Late Pleistocene.

Geochemical analysis

Samples collected in 2022 underwent geochemical analysis from three distinct silt profiles: JN-1, JN-2, and JN-3, in Marten’s and Primitive Men’s Corridors (figure 1). JN-1 and JN-2 were sampled every 20 cm, while JN-3, beneath the tourist route, had 20 samples at 10 cm intervals.

Twenty-two samples from the JN-1 profile in the Marten Corridor were lab-tested. This area features loose diamictonian deposits with boulders, marble, and travertine fragments—signs of calcium carbonate breakdown. Bone material abounds, while quartz and gneiss clasts are scarce. The profile lacks evidence of intermittent water activity, and travertine tile orientation suggests NW gravitational transport, especially in the upper silt layer (figures 1, S1). Six more JN-2 profile samples, below rimstones in Marten’s Corridor (figures 1, S1), were collected at the Primitive Man Corridor juncture. The profile has loose diamicton layered over boulders, with a travertine top. JN-3's silt deposits show cohesive sediments amidst sharp stones, rare bone fragments within a massive diamicton (figures 1, S1).

Silt samples underwent freeze-drying in a laboratory Beta 1-8 LD plus freeze dryer (Martin Christ), followed by homogenization in an agate mortar. Laboratory analyses included: (1) pyrolysis loss on ignition at 550ºC (LOI) for organic matter; (2) total carbon, nitrogen, and sulphur assessment using Element Analyzer CNS – Vario Max CNS; (3) organic carbon quantification (TOC) via Analyzer Rapid CS cube; (4) phosphate content analysis with FIA injection-flow analyser; (5) measurement of the concentration of Na, K, Ca, Mg, Fe, Mn, Cu, Zn, and Pb on the SOILAAR 969 atomic absorption spectrometer (Unicam). Samples were prepared in Teflon bombs using concentrated HNO_3_, HCl, and perhydrol in a Speedwave four microwave mineralizer. JN-3 profile extracts were obtained by agitating with water, then tested for nitrites (NO_2_), nitrates (NO_3_), ammonium (NH_4_), and chlorine using Spectroquant Pharo100 spectrometry (Merck).

Fossil material and collagen extraction

Bone collagen reflects diet before death, while dentine collagen forms during tooth development, affecting δ^15^N. To compare δ^13^C and δ^15^N values across tissues, an adjustment proposed by Bocherens ^5^ was used: δ^13^C_bone_ = δ^13^C_teeth_ + 0.04‰ and δ^15^N_bone_ = δ^15^N_teeth_ - 1.9‰. AMS radiocarbon dating authenticated the geological age of these specimens (Poznań Radiocarbon Laboratory, Poland) (Table S2).

Formerly published data from three Central European caves—Nietoperzowa Cave (438 m a. s. l.), Perspektywiczna Cave (345 m a. s. l.), and Slovakia: Medvedia Cave, (905 m a. s. l.) (Table S3)—were considered. Altitude affects cave bear isotopic signatures, so Krajcarz, et al. ^6^ applied the adjustment: δ^13^C-adj-alt = δ^13^C - (0.0006 x altitude) and δ^15^N-adj-alt = δ^15^N + (0.0013 x altitude), normalizing all data to sea level (i.e., 0 m a.s.l.) for comparison. Isotopic data were compared using an isotopic niche space approach and Kernel Utilisation Density (KUD) model at the 95% contour level, calculated with the rKIN package in R^4^.

Collagen extraction followed a modification of the Longin method, integrating an additional NaOH treatment. The protocol involved bone grinding, followed by treatments: (1) decalcification in HCl, (2) NaOH to remove contaminants, and (3) gelatinization in weak HCl at 80°C for 16 hours. Centrifugation and freeze-drying prepared the solution. Carbon and nitrogen were measured using a Flash 2000 Elemental Analyzer (Thermo Fisher Scientific). The C:N ratio was determined, and samples falling within the range 2.9-3.6 underwent stable carbon and nitrogen isotopic composition measurement using a Delta V Plus IRMS. Ratios were expressed as δ deviations in per mille (‰) from international standards: atmospheric nitrogen for nitrogen and Pee Dee Belemnite (PDB) for carbon, calculated according to the equation: δsample = (R_sample_/R_standard_ − 1) × 1000, where R represents the isotopic ratio, ^15^N/^14^N or ^13^C/^12^C. Precision was <0.1‰ for δ^15^N and <0.2‰ for δ^13^C. Analysis occurred at the Laboratory of Biogeochemistry and Environmental Protection, University of Warsaw (Poland), employing international standards for calibration and precision assurance.

Chronology

Measurements were conducted via the 1.5 SDH-Pelletron Compact Carbon AMS instrument at the Poznań Radiocarbon Laboratory (National Electrostatics Corporation, Poland). Radiocarbon dates were calibrated using OxCal ver. 4.4 software and the IntCal20 curve^7^.

A long bone underwent U-Th dating at the U-series Laboratory, Institute of Geological Sciences, Polish Academy of Sciences, Warsaw^8^. The process involved ultrasonic cleaning, grinding, and sieving with a 0.2 mm mesh size. Collagen assessment for suitable C:N ratios (2.9-3.6) for dating was conducted using an elemental analyser. Cleaning steps followed in HCl and NaOH, extracting the protein phase filtered with 30 kDa ceramic filters. Uranium-thorium isotope separation used DOWEX 1x8, monitored by a ^228^Th-^232^U spike (UDP10030 tracer solution by Isotrac, AEA Technology). Uranium and thorium concentrations were alpha-spectrometrically measured with an OCTETE (Ortec) PC spectrometer. U-series analysis computed ages iteratively based on activity ratios (^230^Th/^234^U, ^234^U/^238^U), using decay constants (^238^U, ^234^U, ^230^Th, ^232^Th) by other authors^9-11^. Samples with low ^230^Th/^232^Th ratios below 20 were adjusted for initial thorium contamination using silicate activity ratio ^230^Th/^232^Th (0.83 ± 0.42), derived from the ^232^Th/^238^U activity ratio (1.21 ± 0.6), ^230^Th/^238^U activity ratio (1.0 ± 0.1), and ^234^U/^238^U activity ratio (1.0 ± 0.1).

**Figure S1.** Sections of Niedźwiedzia Cave’s profiles. (a) The lower section of the JN-1 profile in Marten’s Corridor 1, 1. light-brown sandy-silts, 2. randomly-bedded, dark-brown loam with marble rubble and bones, 3. marble boulders and rubble, 4. isolated fragments of travertine cover, 5. deposit sample, 6. bone sample. (b) The JN-2 profile at the entrance of Marten’s Corridor, 1. light-brown calcareous silty-sand; 2. travertine cover; 3. dark-brown loam with marble rubble and bones; 4. marble boulders and rubble; 5. deposit sample; 6. bone sample. (c) The upper section of the JN3 profile in the Primitive Man’s Corridor. Photos by U. Ratajczak-Skrzatek.

**Figure S2.** The pyrolysis losses (OM) and the content of biogenic elements in the analyzed profiles of sedimentary deposits of Niedźwiedzia Cave.

**Figure S3.** The pyrolysis losses (OM) and the content of selected elements in the sedimentary deposits of Niedźwiedzia Cave.

**Figure S4.** The JN3 profile – content of nitrites, nitrates, chlorides, and urea in water extracts from sedimentary deposits.

**Figure S5.** Niedźwiedzia Cave’s isotopic niche compared to those of Nietoperzowa, Perspektywiczna and Medvedia Caves. Data used for this plot are in Table S3.

**Figure S6.** The system of correlational relationships between the analysed biogenic elements and selected metals.

**Table S1.** Dental wear stages and corresponding ontogenetic ages in years^1^.

**Table S2.** List of cave bear fossil samples from Niedźwiedzia Cave (altitude 800 m a.s.l.) with isotopic content and direct radiocarbon dating.

**Table S3.** Stable isotopic data of cave bear samples from Niedźwiedzia Cave and different sites in Central Europe obtained from literature sources. Adjusted δ^13^C-adj-alt and δ^15^N-adj-alt with the use of altitudinal gradients given by ^6^.

**References**

1. Stiner, M. C. Mortality analysis of Pleistocene bears and its paleoanthropological relevance. *J. Hum. Evol.* **34**, 303-326, doi:10.1006/jhev.1997.0198 (1998).

2. Lyman, R. L. *Vertebrate taphonomy*. (University Press, 1994).

3. Weinstock, J. Epiphyseal fusion in brown bears: a population study of grizzlies (*Ursus arctos horribilis*) from Montana and Wyoming. *Int. J. Osteoarchaeol.* **19**, 416-423, doi:10.1002/oa.980 (2009).

4. R_Core_Team. R: A language and environment for statistical computing. R Foundation for Statistical Computing, Vienna, Austria. <https://www.R-project.org/>. (2022).

5. Bocherens, H. Isotopic tracking of large carnivore palaeoecology in the mammoth steppe. *Quat. Sci. Rev.* **117**, 42-71, doi:10.1016/j.quascirev.2015.03.018 (2015).

6. Krajcarz, M. *et al.* Isotopic variability of cave bears (δ15N, δ13C) across Europe during MIS 3. *Quat. Sci. Rev.* **131**, 51-72, doi:10.1016/j.quascirev.2015.10.028 (2016).

7. Reimer, P. J. *et al.* The Intcal20 Northern Hemisphere Radiocarbon Age Calibration Curve (0-55 Cal Kbp). *Radiocarbon* **62**, 725-757, doi:10.1017/Rdc.2020.41 (2020).

8. Baca, M. *et al.* Ancient DNA and dating of cave bear remains from Niedźwiedzia Cave suggest early appearance of *Ursus ingressus* in Sudetes. *Quat. Int.* **339**, 217-223, doi:10.1016/j.quaint.2013.08.033 (2014).

9. Cheng, H. *et al.* Improvements in Th dating, Th and U half-life values, and U-Th isotopic measurements by multi-collector inductively coupled plasma mass spectrometry. *Earth Planet. Sci. Lett.* **371-372**, 82-91, doi:10.1016/j.epsl.2013.04.006 (2013).

10. Jaffey, A. H., Flynn, K. F., Glendenin, L. E., Bentley, W. C. & Essling, A. M. Precision Measurement of Half-Lives and Specific Activities of U-235 and U-238. *Phys. Rev. C* **4**, 1889-1906, doi:10.1103/PhysRevC.4.1889 (1971).

11. Holden, N. E. Total half-lives for selected nuclides. *Pure Appl. Chem.* **62**, 941-958, doi:10.1351/pac199062050941 (1990).
