# Supplementary material for: Fate and preservation of the Late Pleistocene cave bears from Niedźwiedzia Cave in Poland, through taphonomy, pathology, and geochemistry
Source: Sci Rep. 2024 Apr 29;14:9775. doi: 10.1038/s41598-024-60222-3 (PMC11059340; doi:10.1038/s41598-024-60222-3)
Supplement: Supplementary file 8 — Supplementary Table S1. [file 41598_2024_60222_MOESM8_ESM.pdf]

Table S1. Dental wear stages vs ontogenetic ages in years.

Dental wear stages	Ontogenetic ages		
	From	To	Mean
I	0	1.5	0.75
II	1.5	3	2.5
III	3	4	3.5
IV	4	8	6
V	8	12	10
VI	12	16	14
VII	16	20	18
VIII	20	24	22
IX	24	27	25.5
