# Supplementary material for: Fate and preservation of the Late Pleistocene cave bears from Niedźwiedzia Cave in Poland, through taphonomy, pathology, and geochemistry
Source: Sci Rep. 2024 Apr 29;14:9775. doi: 10.1038/s41598-024-60222-3 (PMC11059340; doi:10.1038/s41598-024-60222-3)
Supplement: Supplementary file 9 — Supplementary Table S2. [file 41598_2024_60222_MOESM9_ESM.pdf]

Table S2. List of cave bear fossil samples from Niedziwiedzia Cave with isotopic results and direct radiocarbon dating (* - tooth values recalculated to bone equivalent)

Lab No.	Site	altitude	Country	Level	Skeletal element	Excavation/Sample No.	direct date (^{14}C BP)	Collagen yield mg/g	$\%C_{coll}$	$\%N_{coll}$	C/N	$\delta^{13}C$	$\delta^{15}N$
ursK-47	Niedźwiedzia	800	Poland	14	cervical vertebra	JNK/Ui/4473		95,49	41,74	14,9	3,27	-20,87	2,59
ursK-54	Niedźwiedzia	800	Poland	14	tibia	JNK/Ui/5340		76,14	39,31	13,9	3,3	-22,12	1,11
ursK-58	Niedźwiedzia	800	Poland	8	tibia	JNK/UI/22621		65,09	40,42	14,37	3,28	-22,6	3,09
ursK-16	Niedźwiedzia	800	Poland	14	lumbar vertebra	JNK/Ui/17720		91,17	41,87	14,94	3,27	-21,26	2,84
ursK-23	Niedźwiedzia	800	Poland	14	pelvis	JNK/Ui/17927		82,54	39,73	13,99	3,31	-23,99	6,51
ursK-24	Niedźwiedzia	800	Poland	16	fibula	JNK/Ui/5911		101,79	40,74	14,59	3,26	-21,78	2,33
ursK-25	Niedźwiedzia	800	Poland	2	fibula	JNK/UI/26665		99,39	41,34	14,78	3,26	-21,85	1,92
ursK-26	Niedźwiedzia	800	Poland	22	lumbar vertebra	JNK/Ui/17717		95,15	40,87	15,08	3,16	-20,93	1,71
ursK-30	Niedźwiedzia	800	Poland	19	lumbar vertebra	JNK/Ui/4996		82,69	42,81	15,32	3,26	-21,86	2,95
ursK-36	Niedźwiedzia	800	Poland	16	ulna	JNK/Ui/26761		71,56	42,41	15,1	3,28	-21,57	1,77
ursK-38	Niedźwiedzia	800	Poland	4	lumbar vertebra	JNK/Ui/16017		116,39	39,99	14,01	3,33	-22,46	1,83
ursK-44	Niedźwiedzia	800	Poland	17	rib	JNK/Ui/26911		127,43	40,63	14,52	3,26	-22,52	3,26
ursK-28	Niedźwiedzia	800	Poland	25	lumbar vertebra	JNK/Ui/4613		98,24	41,09	14,7	3,26	-21,22	2,44
ursK-34	Niedźwiedzia	800	Poland	6	lumbar vertebra	JNK/Ui/144678		98,06	40,59	14,06	3,37	-22,58	1,41
ursK-39	Niedźwiedzia	800	Poland	8	thoracic vertebra	JNK/Ui/16083		69,2	41,97	15,09	3,25	-21,05	2,21
ursK-43	Niedźwiedzia	800	Poland	14	rib	JNK/Ui/26914		63,85	37,44	13,21	3,31	-21,5	1,79
ursK-53	Niedźwiedzia	800	Poland	20	C1*	JNK/Ui/19150		31,15	40,28	13,92	3,38	-22,64	2,36
ursK-69	Niedźwiedzia	800	Poland	18	I3*	JNK/Ui/25754		60,05	34,9	12,04	3,38	-22,38	2,31
ursK-14	Niedźwiedzia	800	Poland	19	C1*	JNK/Ui/26801		43,33	39,11	14,11	3,23	-22,13	1,85
ursK-21	Niedźwiedzia	800	Poland	11	pelvis	JNK/Ui/23033		101,61	40,67	14,36	3,3	-22,81	3,12
ursK-JN1(40	Niedźwiedzia	800	Poland		long bone		43000±2000 BP	112,85	41,58	14,98	3,24	-22,64	2,43
ursK-JN1(S3	Niedźwiedzia	800	Poland		long bone		>45000 BP	105,79	42,07	14,67	3,35	-21,17	1,37
ursK-JN2(40	Niedźwiedzia	800	Poland		long bone		>46000 BP	110,33	42,44	14,76	3,35	-21,94	1,78
ursK-37	Niedźwiedzia	800	Poland	6	ulna	JNK/Ui/4081		88,61	41,98	14,96	3,27	-22,86	4,08
ursK-48*	Niedźwiedzia	800	Poland	18	C1	JNK/Ui/15577		59,2	40,2	14,34	3,27	-23,82	2,83
ursK-67	Niedźwiedzia	800	Poland	21	tibia	JNK/Ui/26871		80,02	40,18	13,87	3,38	-21,49	1,68
ursK-18	Niedźwiedzia	800	Poland	5	skull	JNK/Ui/26895		87,17	40,09	13,93	3,36	-21,83	3,53
ursK-19	Niedźwiedzia	800	Poland	6	lumbar vertebra	JNK/Ui/25124		96,69	38,84	13,88	3,26	-20,59	2,41
ursK-20	Niedźwiedzia	800	Poland	24	lumbar vertebra	JNK/Ui/16234		127,6	41,01	14,48	3,3	-19,95	7,2
ursK-32	Niedźwiedzia	800	Poland	6	ulna	JNK/Ui/25126		99,93	41,34	14,27	3,38	-20,34	1,69
ursK-31	Niedźwiedzia	800	Poland	3	pelvis	JNK/Ui/26885		46,2	38,01	13,5	3,28	-21,83	2,13
ursK-40	Niedźwiedzia	800	Poland	12	lumbar vertebra	JNK/Ui/24710		50,77	37,58	12,82	3,42	-21,48	1,26
ursK-46	Niedźwiedzia	800	Poland	2	pelvis	JNK/Ui/24664		93,2	43	15,26	3,29	-21,42	1,95
ursK-59*	Niedźwiedzia	800	Poland	6	I2	JNK/Ui/25370		48,12	37,76	13,43	3,28	-23,53	5,6
ursK-63*	Niedźwiedzia	800	Poland	6	I2	JNK/Ui/25372		97,41	40,84	14,23	3,35	-22,49	4,06
ursK-64	Niedźwiedzia	800	Poland	13	ulna	JNK/Ui/26246		69,59	39,33	13,86	3,31	-21,27	1,88
ursK-72	Niedźwiedzia	800	Poland	10	fibula	JNK/Ui/18097		54,24	35,26	12,49	3,29	-21,3	1,27
ursK-5(D)*	Niedźwiedzia	800	Poland		teeth			97,46	42,2	14,91	3,3	-22,3	2,22
ursK-7(F)*	Niedźwiedzia	800	Poland		teeth			85,02	40,92	14,3	3,34	-20,83	4,08
