# Supplementary material for: Fate and preservation of the Late Pleistocene cave bears from Niedźwiedzia Cave in Poland, through taphonomy, pathology, and geochemistry
Source: Sci Rep. 2024 Apr 29;14:9775. doi: 10.1038/s41598-024-60222-3 (PMC11059340; doi:10.1038/s41598-024-60222-3)
Supplement: Supplementary file 10 — Supplementary Table S3. [file 41598_2024_60222_MOESM10_ESM.pdf]

Table S3 Stable isotopic data of cave bear samples from Niedźwiedzia Cave and different sites in Central Europe obtained from literature sources. Adjusted δ^{13}C-adj-alt and δ^{15}N-adj-alt with use of altitudinal gradients given by Krajcarz et al. 2016

Lab No.	Site	altitude	Country	δ^{13}Craw_data	δ^{15}Nraw_data	δ^{13}Cadj-alt	δ^{15}Nadj-alt
ursK-47	Niedźwiedzia Cave	800	Poland	-20,87	2,59	-21,35	3,63
ursK-54	Niedźwiedzia Cave	800	Poland	-22,12	1,11	-22,6	2,15
ursK-58	Niedźwiedzia Cave	800	Poland	-22,6	3,09	-23,08	4,13
ursK-16	Niedźwiedzia Cave	800	Poland	-21,26	2,84	-21,74	3,88
ursK-23	Niedźwiedzia Cave	800	Poland	-23,99	6,51	-24,47	7,55
ursK-24	Niedźwiedzia Cave	800	Poland	-21,78	2,33	-22,26	3,37
ursK-25	Niedźwiedzia Cave	800	Poland	-21,85	1,92	-22,33	2,96
ursK-26	Niedźwiedzia Cave	800	Poland	-20,93	1,71	-21,41	2,75
ursK-30	Niedźwiedzia Cave	800	Poland	-21,86	2,95	-22,34	3,99
ursK-36	Niedźwiedzia Cave	800	Poland	-21,57	1,77	-22,05	2,81
ursK-38	Niedźwiedzia Cave	800	Poland	-22,46	1,83	-22,94	2,87
ursK-44	Niedźwiedzia Cave	800	Poland	-22,52	3,26	-23	4,3
ursK-28	Niedźwiedzia Cave	800	Poland	-21,22	2,44	-21,7	3,48
ursK-34	Niedźwiedzia Cave	800	Poland	-22,58	1,41	-23,06	2,45
ursK-39	Niedźwiedzia Cave	800	Poland	-21,05	2,21	-21,53	3,25
ursK-43	Niedźwiedzia Cave	800	Poland	-21,5	1,79	-21,98	2,83
ursK-53	Niedźwiedzia Cave	800	Poland	-22,64	2,36	-23,12	3,4
ursK-69	Niedźwiedzia Cave	800	Poland	-22,38	2,31	-22,86	3,35
ursK-14	Niedźwiedzia Cave	800	Poland	-22,13	1,85	-22,61	2,89
ursK-21	Niedźwiedzia Cave	800	Poland	-22,81	3,12	-23,29	4,16
ursK-JN1(40)	Niedźwiedzia Cave	800	Poland	-22,64	2,43	-23,12	3,47
ursK-JN1(S3)	Niedźwiedzia Cave	800	Poland	-21,17	1,37	-21,65	2,41
ursK-JN2(40)	Niedźwiedzia Cave	800	Poland	-21,94	1,78	-22,42	2,82
ursK-37	Niedźwiedzia Cave	800	Poland	-22,86	4,08	-23,34	5,12
ursK-48*	Niedźwiedzia Cave	800	Poland	-23,82	2,83	-24,3	3,87
ursK-67	Niedźwiedzia Cave	800	Poland	-21,49	1,68	-21,97	2,72
ursK-18	Niedźwiedzia Cave	800	Poland	-21,83	3,53	-22,31	4,57
ursK-19	Niedźwiedzia Cave	800	Poland	-20,59	2,41	-21,07	3,45
ursK-20	Niedźwiedzia Cave	800	Poland	-19,95	7,2	-20,43	8,24
ursK-32	Niedźwiedzia Cave	800	Poland	-20,34	1,69	-20,82	2,73
ursK-31	Niedźwiedzia Cave	800	Poland	-21,83	2,13	-22,31	3,17
ursK-40	Niedźwiedzia Cave	800	Poland	-21,48	1,26	-21,96	2,3
ursK-46	Niedźwiedzia Cave	800	Poland	-21,42	1,95	-21,9	2,99
ursK-59*	Niedźwiedzia Cave	800	Poland	-23,53	5,6	-24,01	6,64
ursK-63*	Niedźwiedzia Cave	800	Poland	-22,49	4,06	-22,97	5,1
ursK-64	Niedźwiedzia Cave	800	Poland	-21,27	1,88	-21,75	2,92
ursK-72	Niedźwiedzia Cave	800	Poland	-21,3	1,27	-21,78	2,31
ursK-5(D)*	Niedźwiedzia Cave	800	Poland	-22,3	2,22	-22,78	3,26
ursK-7(F)*	Niedźwiedzia Cave	800	Poland	-20,83	4,08	-21,31	5,12
MJ-1	Medvedja Cave	905	Slovakia	-20,7	1,4	-21,243	2,5765
MJ-2	Medvedja Cave	905	Slovakia	-21	1,9	-21,543	3,0765
MJ-3	Medvedja Cave	905	Slovakia	-21	3,1	-21,543	4,2765
MJ-4	Medvedja Cave	905	Slovakia	-20,9	1,9	-21,443	3,0765
MJ-5	Medvedja Cave	905	Slovakia	-21	2,3	-21,543	3,4765
MJ-6	Medvedja Cave	905	Slovakia	-21	2,2	-21,543	3,3765
MJ-7	Medvedja Cave	905	Slovakia	-21,3	1,5	-21,843	2,6765
MJ-8	Medvedja Cave	905	Slovakia	-21,1	1,5	-21,643	2,6765
MJ-9	Medvedja Cave	905	Slovakia	-21	1,9	-21,543	3,0765
MJ-10	Medvedja Cave	905	Slovakia	-20,7	3,7	-21,243	4,8765
MJ-11	Medvedja Cave	905	Slovakia	-20,9	2,8	-21,443	3,9765
MJ-12	Medvedja Cave	905	Slovakia	-20,8	0,8	-21,343	1,9765
MJ-13	Medvedja Cave	905	Slovakia	-21,4	1,8	-21,943	2,9765
MJ-14	Medvedja Cave	905	Slovakia	-21	2,3	-21,543	3,4765
MJ-15	Medvedja Cave	905	Slovakia	-20,8	1,1	-21,343	2,2765
MJ-16	Medvedja Cave	905	Slovakia	-20,7	0,8	-21,243	1,9765
MJ-17	Medvedja Cave	905	Slovakia	-21,2	1,8	-21,743	2,9765
MJ-18	Medvedja Cave	905	Slovakia	-21	1,7	-21,543	2,8765
MJ-19	Medvedja Cave	905	Slovakia	-20,8	1,3	-21,343	2,4765
MJ-20	Medvedja Cave	905	Slovakia	-20,9	1,6	-21,443	2,7765
IN-01	Nietoperzowa Cave	438	Poland	-22,1	5,1	-22,3628	5,6694
IN-03	Nietoperzowa Cave	438	Poland	-20,5	2,5	-20,7628	3,0694
IN-09	Nietoperzowa Cave	438	Poland	-21,8	2,4	-22,0628	2,9694
IN-15	Nietoperzowa Cave	438	Poland	-21,3	2,5	-21,5628	3,0694
IN-21	Nietoperzowa Cave	438	Poland	-21,2	3,6	-21,4628	4,1694
IN-22	Nietoperzowa Cave	438	Poland	-21	2,1	-21,2628	2,6694
IN-24	Nietoperzowa Cave	438	Poland	-21,2	2,7	-21,4628	3,2694
IN-25	Nietoperzowa Cave	438	Poland	-21,2	1,4	-21,4628	1,9694
IN-26	Nietoperzowa Cave	438	Poland	-21,1	3,8	-21,3628	4,3694
IN-29	Nietoperzowa Cave	438	Poland	-21,2	2,7	-21,4628	3,2694
IN-30	Nietoperzowa Cave	438	Poland	-23,2	2,8	-23,4628	3,3694
IN-31	Nietoperzowa Cave	438	Poland	-21,1	2,1	-21,3628	2,6694
IN-46	Nietoperzowa Cave	438	Poland	-21,3	1,7	-21,5628	2,2694
IN-49	Nietoperzowa Cave	438	Poland	-21,5	3,9	-21,7628	4,4694
IN-54	Nietoperzowa Cave	438	Poland	-21,3	3	-21,5628	3,5694
IN-55	Nietoperzowa Cave	438	Poland	-21,8	4,2	-22,0628	4,7694
IN-56	Nietoperzowa Cave	438	Poland	-21,2	2,1	-21,4628	2,6694
IN-60	Nietoperzowa Cave	438	Poland	-21,2	2,8	-21,4628	3,3694
IP-05	Perspektywiczna Cave	345	Poland	-21,2	1,9	-21,407	2,3485
IP-06	Perspektywiczna Cave	345	Poland	-21,5	5,1	-21,707	5,5485
IP-09	Perspektywiczna Cave	345	Poland	-21,4	3,3	-21,607	3,7485
IP-13	Perspektywiczna Cave	345	Poland	-21,3	4,1	-21,507	4,5485
IP-14	Perspektywiczna Cave	345	Poland	-21,2	3	-21,407	3,4485
IP-15	Perspektywiczna Cave	345	Poland	-21,1	2,3	-21,307	2,7485
IP-16	Perspektywiczna Cave	345	Poland	-21,9	2,7	-22,107	3,1485
IP-17	Perspektywiczna Cave	345	Poland	-21,7	4,1	-21,907	4,5485
IP-18	Perspektywiczna Cave	345	Poland	-21,2	2,4	-21,407	2,8485
IP-19	Perspektywiczna Cave	345	Poland	-21	1,6	-21,207	2,0485
